# Supplementary material for: Identification of risk factors associated with prolonged hospital stay following primary knee replacement surgery: a retrospective, longitudinal observational study
Source: BMJ Open. 2022 Dec 16;12(12):e068252. doi: 10.1136/bmjopen-2022-068252 (PMC9764602; doi:10.1136/bmjopen-2022-068252)

Supplementary material

Supplementary Table S1: OPCS-4 codes used to identify primary hip and knee replacement operations

Supplementary Table S2: Sample characteristics for total sample (N=3008), those with complete data and with a previous admission (N=1221) and those with complete data and no previous admission (N=2295)

Supplementary Table S3: Multivariable models for binary length of stay outcome (admissions > 7 days)

Supplementary Figure S1: Calibration plot for binary length of stay outcome (full regression model)

Supplementary Figure S2: Calibration plot for binary length of stay outcome (backwards selection regression model)

Supplementary Table S4: Multivariable models for continuous length of stay outcome (≤ 30 days)

Supplementary Table S5: Multivariable models for medically fit for discharge date outcome (MFFD date < discharge date)

Supplementary Figure S3: Calibration plot for medically fit for discharge (full regression model)

Supplementary Figure S4: Calibration plot for medically fit for discharge (backwards selection regression model)

Supplementary Table S1: OPCS-4 codes used to identify primary hip and knee replacement operations

Category	Code	Description	Notes
Primary Total Knee Replacement	W40.1	Primary total prosthetic replacement of knee joint using cement	
	W40.8	Other specified total prosthetic replacement of knee joint using cement	
	W40.9	Unspecified total prosthetic replacement of knee joint using cement	
	W41.1	Primary total prosthetic replacement of knee joint not using cement	
	W41.8	Other specified total prosthetic replacement of knee joint not using cement	
	W41.9	Unspecified total prosthetic replacement of knee joint not using cement	
	W42.1	Primary total prosthetic replacement of knee joint NEC	
	W42.8	Other specified other total prosthetic replacement of knee joint	
	W42.9	Unspecified other total prosthetic replacement of knee joint	
	O18.1	Primary hybrid prosthetic replacement of knee joint using cement	
	O18.8	Other specified hybrid prosthetic replacement of knee joint using cement	
	O18.9	Unspecified hybrid prosthetic replacement of knee joint using cement	
Resurfacing / Reconstruction	W58.1	Primary resurfacing arthroplasty of joint	Require combination with site + combination codes to ID
	W58.8	Other specified reconstruction of joint	Require combination with site + combination codes to ID
	W58.9	Unspecified other reconstruction of joint	Require combination with site + combination codes to ID
Primary unicondylar / unicompartmental knee operations	W52.1	Primary prosthetic replacement of articulation of bone using cement NEC	Require combination with site + combination codes to ID
	W52.8	Other specified prosthetic replacement of articulation of other bone using cement	Require combination with site + combination codes to ID

	W52.9	Unspecified prosthetic replacement of articulation of other bone using cement	Require combination with site + combination codes to ID
	W53.1	Primary prosthetic replacement of articulation of bone not using cement NEC	Require combination with site + combination codes to ID
	W53.9	Unspecified prosthetic replacement of articulation of other bone not using cement	Require combination with site + combination codes to ID
	W54.0	Conversion from previous prosthetic replacement of articulation of bone NEC	Require combination with site + combination codes to ID
	W54.1	Primary prosthetic replacement of articulation of bone NEC	Require combination with site + combination codes to ID
	W54.8	Other specified other prosthetic replacement of articulation of other bone	Require combination with site + combination codes to ID
	W54.9	Unspecified other prosthetic replacement of articulation of other bone	Require combination with site + combination codes to ID

Supplementary Table S2: Sample characteristics for total sample (N=3008), those with complete data and with a previous admission (N=1221) and those with complete data and no previous admission (N=2295)

Main characteristics		Total sample N=3008	Complete case sample included in multivariable model with continuous days since last discharge variable N=1221	Complete case sample included in multivariable model with categorical days since last discharge variable N=2295
Age, mean (SD)		67.5 (11.4)	68.0 (11.2)	67.4 (11.3)
Sex, N (%)	Female	1803 (59.9)	734 (60.1)	1381 (60.2)
	Male	1205 (40.1)	487 (39.9)	914 (39.8)
IMD score, N (%)	1 (least deprived)	753 (25.0)	310 (25.4)	588 (25.6)
	2	719 (23.9)	297 (24.3)	564 (24.6)
	3	515 (17.1)	225 (18.4)	395 (17.2)
	4	538 (17.9)	212 (17.4)	418 (18.2)
	5 (most deprived)	426 (14.2)	177 (14.5)	330 (14.4)
	(missing)	57 (1.9)	-	-
Ethnicity, N (%)	Non-White	90 (3.0)	47 (3.9)	76 (3.3)
	White	1827 (60.7)	779 (63.8)	1368 (59.6)
	(missing)	1091 (36.3)	395 (32.4)	851 (37.1)
Comorbidities, N (%)	0	1681 (55.9)	618 (50.6)	1260 (54.9)
	1-2	1126 (37.4)	485 (39.7)	867 (37.8)
	3-4	175 (5.8)	101 (8.3)	148 (6.5)
	>=5	26 (0.9)	17 (1.4)	20 (0.9)

Supplementary Table S3: Multivariable models for binary length of stay outcome (admissions > 7 days) (N=2295)

Variable	Multivariable logistic regression (no variable selection)		Lasso regression	Elastic net logistic regression	Ridge regression	Multivariable logistic regression (backwards selection)	
	OR (95%CI)	p	OR	OR	OR	OR (95%CI)	p
**Age at admission**	1.06 (1.04 to 1.07)	<0.001	1.78	1.77	1.70	1.06 (1.04 to 1.07)	<0.001
**Sex (female vs male)**	0.91 (0.71 to 1.17)	0.470	0.98	0.98	0.97	-	
**Deprivation (IMD score)**							
1 (least deprived)	*1.00*		*1.00*	*1.00*	*1.00*	*1.00*	
2	1.74 (1.22 to 2.48)	0.002	1.20	1.20	1.19	1.74 (1.22 to 2.47)	0.002
3	1.76 (1.19 to 2.60)	0.005	1.17	1.16	1.16	1.74 (1.18 to 2.58)	0.005
4	1.55 (1.04 to 2.29)	0.030	1.12	1.12	1.12	1.54 (1.04 to 2.28)	0.030
5 (most deprived)	2.05 (1.36 to 3.09)	0.001	1.21	1.21	1.20	2.03 (1.34 to 3.05)	0.001
**Comorbidities (weighted Charlson index)**							
0	*1.00*		*1.00*	*1.00*	*1.00*	*1.00*	
1-2	1.40 (1.08 to 1.81)	0.011	1.15	1.15	1.16	1.40 (1.08 to 1.81)	0.011
3-4	1.86 (1.20 to 2.87)	0.005	1.15	1.15	1.16	1.85 (1.20 to 2.86)	0.006
>=5	2.96 (1.11 to 7.90)	0.030	1.10	1.10	1.11	3.03 (1.14 to 8.05)	0.026
**Time since Last Discharge**							

0-2 months	*1.00*		*1.00*	*1.00*	*1.00*	*1.00*	
2-12 months	0.66 (0.47 to 0.94)	0.023	0.89	0.89	0.91	0.67 (0.47 to 0.95)	0.025
12 months or more	0.40 (0.27 to 0.60)	<0.001	0.75	0.75	0.77	0.41 (0.27 to 0.61)	<0.001
never	0.38 (0.27 to 0.53)	0.761	0.65	0.66	0.68	0.38 (0.28 to 0.53)	<0.001
**Emergency over elective admissions ratio**	0.65 (0.06 to 7.72)	0.736	-	-	0.98	-	
**NEL admissions**	1.00 (0.99 to 1.01)	0.729	-	-	1.03	-	
**NEL occupied beds**	0.999 (0.995 to 1.004)	0.761	1.01	1.01	1.03	-	
**Admission hour category**							
06.00-12.00	*1.00*		*1.00*	*1.00*	*1.00*	*1.00*	
12.00-18.00	1.82 (1.23 to 2.68)	0.003	1.17	1.17	1.16	1.81 (1.23 to 2.66)	0.003
18.00-06:00	76.72 (7.91 to 744.34)	<0.001	1.26	1.26	1.25	77.21 (8.01 to 744.20)	<0.001
**Year of Admission**							
2016	*1.00*		*1.00*	*1.00*	*1.00*	*1.00*	
2017	1.70 (0.92 to 3.17)	0.093	1.16	1.16	1.12	1.66 (0.96 to 2.86)	0.071
2018	2.07 (1.05 to 4.08)	0.036	1.24	1.24	1.19	1.96 (1.12 to 3.42)	0.018
2019	1.21 (0.54 to 2.70)	0.639	-	1.00	0.94	1.14 (0.65 to 1.99)	0.658
**Day of the Week of Admission**							
Sunday							
Monday	*1.00*		*1.00*	*1.00*	*1.00*	*1.00*	

Tuesday	0.88 (0.62 to 1.25)	0.479	-	1.00	0.98	0.89 (0.62 to 1.26)	0.497
Wednesday	0.59 (0.39 to 0.87)	0.008	0.87	0.87	0.86	0.59 (0.40 to 0.88)	0.009
Thursday	0.65 (0.44 to 0.96)	0.031	0.91	0.90	0.89	0.66 0.45 to 0.97)	0.033
Friday	0.76 (0.52 to 1.12)	0.164	0.95	0.95	0.93	0.78 (0.54 to 1.13)	0.185
Saturday	0.21 (0.05 to 0.79)	0.021	0.84	0.84	0.85	0.20 (0.06 to 0.73)	0.014
**Season of Admission**							
Winter (Dec-Feb)	*1.00*		*1.00*	*1.00*	*1.00*	*1.00*	
Spring (Mar-May)	1.11 (0.77 to 1.62)	0.571	1.07	1.07	1.08	1.14 (0.80 to 1.61)	0.479
Summer (Jun-Aug)	0.70 (0.45 to 1.10)	0.121	0.91	0.91	0.91	0.73 (0.50 to 1.06)	0.102
Autumn (Sep-Nov)	0.87 (0.59 to 1.29)	0.490	0.98	0.98	0.96	0.90 (0.64 to 1.28)	0.562
C-statistic	0.73 (0.71 to 0.76)					0.72 (0.69 to 0.74)	

Fig S1: Calibration plot for length of stay (full regression model)

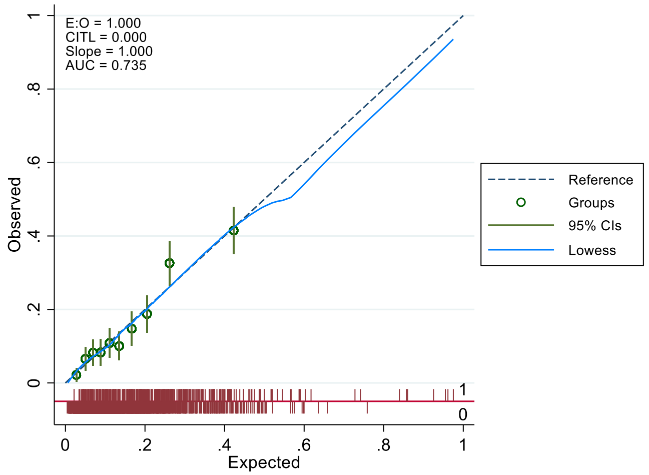

Fig S2: Calibration plot for length of stay (backwards selection regression model)

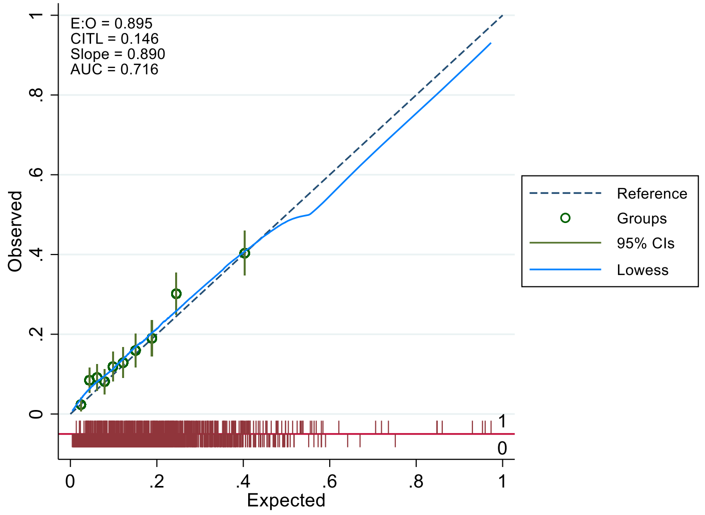

Supplementary Table S4: Multivariable models for continuous length of stay outcome (≤ 30 days) (N=2274)

Variable	Multivariable linear regression (no variable selection)		Lasso regression	Elastic net logistic regression	Ridge regression	Multivariable linear regression (backwards selection)	
	**Coef (95%CI)**	**p**	**Coef**	**Coef**	**Coef**	**Coef (95%CI)**	**p**
**Age at admission**	0.08 (0.07 to 0.09)	<0.001	0.87	0.87	0.83	0.08 (0.07 to 0.09)	<0.001
**Sex (female vs male)**	0.36 (0.05 to 0.66)	0.021	0.15	0.15	0.17	0.36 (0.06 to 0.67)	0.018
**Deprivation (IMD score)**							
1 (least deprived)	*0.00*		*0.00*	*0.00*	*0.00*	*0.00*	
2	0.62 (0.22 to 1.01)	0.002	0.18	0.18	0.20	0.65 (0.26 to 1.04)	0.001
3	0.81 (0.32 to 1.29)	0.001	0.22	0.22	0.24	0.80 (0.32 to 1.28)	0.001
4	0.73 (0.32 to 1.15)	0.001	0.20	0.20	0.22	0.75 (0.33 to 1.16)	<0.001
5 (most deprived)	0.96 (0.46 to 1.47)	<0.001	0.26	0.26	0.27	0.98 (0.47 to 1.48)	<0.001
**Comorbidities (weighted Charlson index)**							
0	*0.00*		*0.00*	*0.00*	*0.00*	*0.00*	
1-2	0.62 (0.31 to 0.93)	<0.001	0.27	0.27	0.28	0.63 (0.31 to 0.94)	<0.001
3-4	1.36 (0.50 to 2.23)	0.002	0.32	0.32	0.32	1.42 (0.55 to 2.29)	0.001
>=5	2.44 (0.12 to 4.75)	0.039	0.20	0.20	0.22	2.48 (0.15 to 4.81)	0.037
**Time since Last Discharge**							

0-2 months	*0.00*		*0.00*	*0.00*	*0.00*	*0.00*	
2-12 months	-0.65 (-1.29 to -0.01)	0.046	-0.15	-0.15	-0.13	-0.65 (-1.29 to -0.02)	0.044
12 months or more	-1.48 (-2.09 to -0.87)	<0.001	-0.46	-0.46	-0.42	-1.45 (-2.06 to -0.84)	<0.001
never	-1.50 (-2.06 to -0.94)	<0.001	-0.62	-0.62	-0.57	-1.49 (-2.05 to -0.93)	<0.001
**Emergency over elective admissions ratio**	-0.19 (-3.34 to 2.95)	0.904	-	-	-0.001	-	
**NEL admissions**	-0.01 (-0.02 to 0.01)	0.732	-	-	-0.05	-	
**NEL occupied beds**	-0.0005 (-0.0066 to 0.0057)	0.884	-	-	0.03	-	
**Admission hour category**							
06.00-12.00	*0.00*		*0.00*	*0.00*	*0.00*	*0.00*	
12.00-18.00	0.87 (0.24 to 1.50)	0.007	0.20	0.20	0.21	0.79 (0.17 to 1.41)	0.012
18.00-06:00	7.61 (2.91 to 12.31)	0.002	0.40	0.40	0.40	7.55 (2.85 to 12.24)	0.002
**Year of Admission**							
2016	*0.00*		*0.00*	*0.00*	*0.00*	*0.00*	
2017	0.36 (-0.30 to 1.03)	0.282	0.05	0.05	0.07	0.20 (-0.27 to 0.67)	0.411
2018	0.73 (0.04 to 1.41)	0.037	0.17	0.17	0.21	0.47 (-0.04 to 0.99)	0.073
2019	0.07 (-0.82 to 0.96)	0.879	-0.10	-0.10	-0.08	-0.21 (-0.71 to 0.30)	0.425
**Day of the Week of Admission**							

Sunday	-	-					
Monday	*0.00*		*0.00*	*0.00*	*0.00*		
Tuesday	1.11 (-0.34 to 0.56)	0.637	-	-	0.02		
Wednesday	0.13 (-0.37 to 0.64)	0.604	-	-	0.03		
Thursday	0.20 (-0.26 to 0.66)	0.399	0.02	0.02	0.06		
Friday	0.53 (0.05 to 1.01)	0.032	0.14	0.14	0.18		
Saturday	-0.49 (-1.55 to -0.57)	0.365	-0.04	-0.04	-0.06		
**Season of Admission**							
Winter (Dec-Feb)	*0.00*		*0.00*	*0.00*	*0.00*		
Spring (Mar-May)	-0.13 (-0.62 to 0.36)	0.604	-	-	-0.02		
Summer (Jun-Aug)	-0.52 (-1.06 to 0.02)	0.060	-0.14	-0.14	-0.16		
Autumn (Sep-Nov)	-0.10 (-0.60 to 0.40)	0.700	-	-	-0.02		
R^2	0.136					0.130	

Supplementary Table S5: Multivariable models for binary medically fit for discharge date outcome (MFFD date < discharge date) (N=2275)

**Variable**	**Multivariable logistic regression (no variable selection)**		**Lasso regression**	**Elastic net logistic regression**	**Ridge regression**	**Multivariable logistic regression (backwards selection)**	
	**OR (95%CI)**	**p**	**OR**	**OR**	**OR**	**OR (95%CI)**	**p**
**Age at admission**	1.07 (1.05 to 1.09)	<0.001	2.13	2.11	1.95	1.07 (1.05 to 1.09)	<0.001
**Sex (female vs male)**	1.70 (1.17 to 2.45)	0.005	1.28	1.29	1.28	1.71 (1.19 to 2.47)	0.004
**Deprivation (IMD score)**							
1 (least deprived)	*1.00*		*1.00*	*1.00*	*1.00*	*1.00*	
2	1.78 (1.06 to 3.02)	0.031	1.22	1.24	1.19	1.76 (1.05 to 2.96)	0.033
3	1.89 (1.08 to 3.33)	0.026	1.22	1.23	1.18	1.84 (1.05 to 3.23)	0.032
4	2.12 (1.22 to 3.67)	0.007	1.28	1.29	1.24	2.07 (1.20 to 3.57)	0.009
5 (most deprived)	2.23 (1.24 to 4.01)	0.007	1.27	1.28	1.22	2.27 (1.27 to 4.06)	0.006
**Comorbidities (weighted Charlson index)**							
0	*1.00*		*1.00*	*1.00*	*1.00*	*1.00*	
1-2	1.91 (1.32 to 2.74)	0.001	1.35	1.35	1.33	1.89 (1.32 to 2.71)	0.001
3-4	2.40 (1.37 to 4.22)	0.002	1.24	1.25	1.25	2.56 (1.48 to 4.42)	0.001
>=5	-		0.79	0.77	0.83	-	
**Time since Last Discharge**							
0-2 months	*1.00*		*1.00*	*1.00*	*1.00*	*1.00*	

2-12 months	0.94 (0.58 to 1.52)	0.792	-	1.00	1.01	0.97 (0.60 to 1.57)	0.907
12 months or more	0.48 (0.27 to 0.85)	0.012	0.79	0.79	0.82	0.53 (0.30 to 0.92)	0.024
never	0.51 (0.32 to 0.82)	0.005	0.74	0.74	0.76	0.53 (0.33 to 0.84)	0.007
**Emergency over elective admissions ratio**	0.97 (0.02 to 37.81)	0.986	-	-	1.03	-	
**NEL admissions**	0.99 (0.98 to 1.01)	0.230	0.91	0.85	0.87	-	
**NEL occupied beds**	1.00 (1.00 to 1.01)	0.592	1.05	0.07	1.09	-	
**Admission hour category**							
06.00-12.00	*1.00*		*1.00*	*1.00*	*1.00*	*1.00*	
12.00-18.00	1.61 (0.92 to 2.81)	0.096	1.13	1.15	1.14	1.56 (0.91 to 2.66)	0.103
18.00-06:00	8.81 (1.77 to 43.87)	0.008	1.14	1.14	1.14	8.11 (1.74 to 37.75)	0.008
**Year of Admission**							
2016	*1.00*		*1.00*	*1.00*	*1.00*	*1.00*	
2017	2.91 (1.00 to 8.41)	0.049	1.37	1.41	1.20	2.95 (1.14 to 7.64)	0.026
2018	4.37 (1.43 to 13.37)	0.010	1.57	1.64	1.36	3.89 (1.49 to 10.12)	0.005
	2.78 (0.79 to 9.82)	0.112	1.26	1.36	1.11	2.32 (0.88 to 6.09)	0.088
**Day of the Week of Admission**							
Sunday							
Monday	*1.00*		*1.00*	*1.00*	*1.00*		

Tuesday	1.67 (0.99 to 2.82)	0.057	1.19	1.19	1.16		
Wednesday	1.32 (0.75 to 2.32)	0.337	1.08	1.08	1.07		
Thursday	1.04 (0.57 to 1.88)	0.901	-	-	0.99		
Friday	1.68 (0.96 to 2.93)	0.069	1.18	1.19	1.17		
Saturday	0.65 (0.16 to 2.68)	0.548	0.96	0.94	0.95		
**Season of Admission**							
Winter (Dec-Feb)	*1.00*		*1.00*	*1.00*	*1.00*		
Spring (Mar-May)	1.37 (0.78 to 2.44)	0.276	1.11	1.12	1.13		
Summer (Jun-Aug)	1.38 (0.72 to 2.65)	0.327	1.10	1.12	1.13		
Autumn (Sep-Nov)	1.54 (0.85 to 2.76)	0.152	1.14	1.17	1.15		
C-statistic	0.77 (0.74 to 0.81)					0.74 (0.71 to 0.78)	

Fig S3: Calibration plot medically fit for discharge (full regression model)

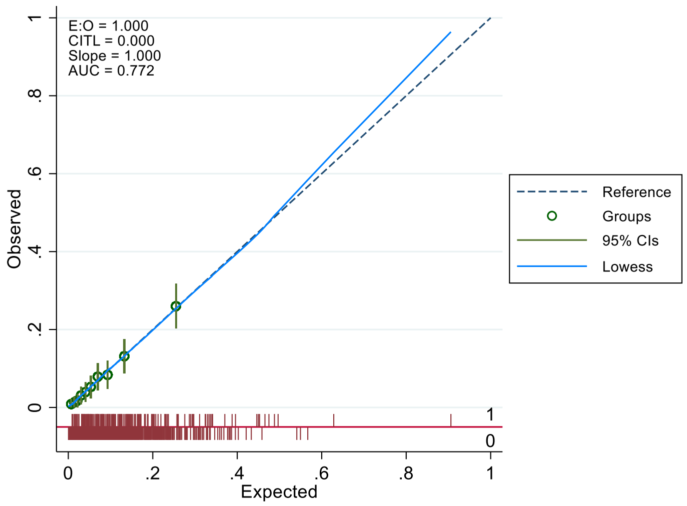

Fig S4: Calibration plot medically fit for discharge (backwards selection model)

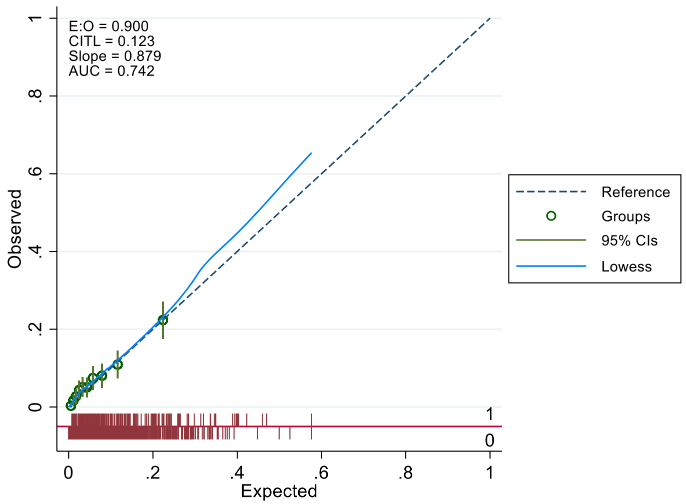

Supplement: Supplementary data [file bmjopen-2022-068252supp001.pdf]
