# Supplementary material for: Identification of risk factors associated with prolonged hospital stay following primary knee replacement surgery: a retrospective, longitudinal observational study
Source: BMJ Open. 2022 Dec 16;12(12):e068252. doi: 10.1136/bmjopen-2022-068252 (PMC9764602; doi:10.1136/bmjopen-2022-068252)
Supplement: Author's manuscript [file bmjopen-2022-068252.draft_revisions.pdf]

BMJ Open

BMJ Open is committed to open peer review. As part of this commitment we make the peer review history of every article we publish publicly available.

When an article is published we post the peer reviewers' comments and the authors' responses online. We also post the versions of the paper that were used during peer review. These are the versions that the peer review comments apply to.

The versions of the paper that follow are the versions that were submitted during the peer review process. They are not the versions of record or the final published versions. They should not be cited or distributed as the published version of this manuscript.

BMJ Open is an open access journal and the full, final, typeset and author-corrected version of record of the manuscript is available on our site with no access controls, subscription charges or pay-per-view fees (http://bmjopen.bmj.com).

If you have any questions on BMJ Open's open peer review process please email info.bmjopen@bmj.com

BMJ Open

Identification of risk factors associated with prolonged hospital stay following primary knee replacement surgery: a longitudinal observational study

Journal:	*BMJ Open*
Manuscript ID	bmjopen-2022-068252
Article Type:	Original research
Date Submitted by the Author:	12-Sep-2022
Complete List of Authors:	wilson, rebecca ; University Hospitals Bristol and Weston NHS Foundation Trust, The National Institute for Health Research Applied Research Collaboration West (NIHR ARC West); University of Bristol, Population Health Sciences, Bristol Medical School<br>Margelyte, Ruta; University of Bristol, Translational Health Sciences, Bristol Medical School<br>Redaniel, Maria Theresa; University Hospitals Bristol and Weston NHS Foundation Trust, The National Institute for Health Research Applied Research Collaboration West (NIHR ARC West); University of Bristol, Population Health Sciences, Bristol Medical School<br>Eyles, Emily; University Hospitals Bristol and Weston NHS Foundation Trust, The National Institute for Health Research Applied Research Collaboration West (NIHR ARC West); University of Bristol, Population Health Sciences, Bristol Medical School<br>Jones, Tim; University Hospitals Bristol and Weston NHS Foundation Trust, The National Institute for Health Research Applied Research Collaboration West (NIHR ARC West); University of Bristol, Population Health Sciences, Bristol Medical School<br>Penfold, Chris; University Hospitals Bristol and Weston NHS Foundation Trust, The National Institute for Health Research Applied Research Collaboration West (NIHR ARC West); University of Bristol, Population Health Sciences, Bristol Medical School<br>Blom, AW; University of Bristol, Translational Health Sciences, Bristol Medical School<br>Elliott, Andrew; North Bristol NHS Trust<br>Harper, Alison; University of Exeter, The National Institute for Health Research Applied Research Collaboration South-West Peninsula (PenARC); University of Exeter, Medical School<br>Keen, Tim; North Bristol NHS Trust<br>Pitt, Martin; University of Exeter, The National Institute for Health Research Applied Research Collaboration South-West Peninsula (PenARC); University of Exeter, Medical School<br>Judge, Andrew; University of Bristol, Translational Health Sciences
Keywords:	Knee < ORTHOPAEDIC & TRAUMA SURGERY, Adult orthopaedics < ORTHOPAEDIC & TRAUMA SURGERY, RHEUMATOLOGY, STATISTICS & RESEARCH METHODS, Orthopaedic & trauma surgery < SURGERY

SCHOLARONE™
Manuscripts

I, the Submitting Author has the right to grant and does grant on behalf of all authors of the Work (as defined in the below author licence), an exclusive licence and/or a non-exclusive licence for contributions from authors who are: i) UK Crown employees; ii) where BMJ has agreed a CC-BY licence shall apply, and/or iii) in accordance with the terms applicable for US Federal Government officers or employees acting as part of their official duties; on a worldwide, perpetual, irrevocable, royalty-free basis to BMJ Publishing Group Ltd ("BMJ") its licensees and where the relevant Journal is co-owned by BMJ to the co-owners of the Journal, to publish the Work in this journal and any other BMJ products and to exploit all rights, as set out in our licence.

The Submitting Author accepts and understands that any supply made under these terms is made by BMJ to the Submitting Author unless you are acting as an employee on behalf of your employer or a postgraduate student of an affiliated institution which is paying any applicable article publishing charge ("APC") for Open Access articles. Where the Submitting Author wishes to make the Work available on an Open Access basis (and intends to pay the relevant APC), the terms of reuse of such Open Access shall be governed by a Creative Commons licence – details of these licences and which Creative Commons licence will apply to this Work are set out in our licence referred to above.

Other than as permitted in any relevant BMJ Author's Self Archiving Policies, I confirm this Work has not been accepted for publication elsewhere, is not being considered for publication elsewhere and does not duplicate material already published. I confirm all authors consent to publication of this Work and authorise the granting of this licence.

For peer review only

Identification of risk factors associated with prolonged hospital stay following primary knee replacement surgery: a longitudinal observational study

Rebecca Wilson, PhD[1,2]; Ruta Margelyte, MSc[3]; Maria Theresa Redaniel, PhD[1,2]; Emily Eyles, PhD[1,2]; Tim Jones, PhD[1,2]; Chris Penfold, PhD[1,2]; Ashley Blom, PhD[3]; Andrew Elliott, MSc[4]; Alison Harper, PhD[5,6]; Tim Keen, MSc[4]; Martin Pitt, EngD[5,6]; Andy Judge, PhD[3]

1. The National Institute for Health Research Applied Research Collaboration West (NIHR ARC West) at University Hospitals Bristol and Weston NHS Foundation Trust, Bristol, UK
2. Population Health Sciences, Bristol Medical School, University of Bristol, Bristol, UK
3. Translational Health Sciences, Bristol Medical School, University of Bristol, Bristol, UK
4. North Bristol NHS Trust, Bristol, UK
5. The National Institute for Health Research Applied Research Collaboration South-West Peninsula (PenARC), University of Exeter Medical School, Exeter, UK
6. Medical School, University of Exeter, Exeter, UK

<u>Corresponding author</u>

Dr Rebecca Wilson

National Institute for Health Research Applied Research Collaboration West (NIHR ARC West)

University Hospitals Bristol NHS Foundation Trust

9th Floor, Whitefriars, Lewins Mead, Bristol, BS1 2NT

For peer review only

rebecca.wilson@bristol.ac.uk

For peer review only

For peer review only - http://bmjopen.bmj.com/site/about/guidelines.xhtml

Abstract

Objectives

To identify risk factors associated with prolonged length of hospital stay and staying in hospital longer than medically necessary following primary knee replacement surgery

Design

Retrospective, longitudinal observational study

Setting

Elective knee replacement surgeries between 2016-2019 were identified using routinely collected data from an NHS Trust in England

Participants

There were 2295 knee replacement patients with complete data included in analysis. The mean age was 68 (standard deviation 11) and 60% were female.

Primary and secondary outcome measures

We assessed a binary length of stay outcome (>7 days), a continuous length of stay outcome (≤30 days) and a binary measure of whether patients remained in hospital when they were medically fit for discharge.

Results

The mean length of stay was 5.0 days (SD 3.9), 15.4% of patients were in hospital for >7 days and 7.1% remained in hospital when they were medically fit for discharge. Longer length of stay was associated with older age (b=0.08, 95% CI 0.07 to 0.09), female sex (b=0.36, 95% CI 0.06 to 0.67), high deprivation (b=0.98, 95% CI 0.47 to 1.48) and more comorbidities (b=2.48, 95% CI 0.15 to 4.81). Remaining in hospital beyond being medically fit for discharge was associated with older age

For peer review only - http://bmjopen.bmj.com/site/about/guidelines.xhtml

(OR=1.07, 95% CI 1.05 to 1.09), female sex (OR=1.71, 95% CI 1.19 to 2.47) and high deprivation (OR=2.27, 95% CI 1.27 to 4.06).

Conclusions

The regression models could be used to identify which patients are likely to occupy hospital beds for longer. This could be helpful in scheduling operations to aid hospital efficiency by planning these patients' operations for when the hospital is less busy.

For peer review only

Strengths and limitations (no more than 5 bullet points)

- Robust statistical methods used to develop statistical models with good discrimination and calibration
- Consistent findings across three output measures of hospital throughput
- Single centre study may restrict generalisability of findings
- Data do not include operations during or post the COVID-19 pandemic, after which the healthcare landscape changed and waiting list times have increased further

Word count: 2367

For peer review only - http://bmjopen.bmj.com/site/about/guidelines.xhtml

Introduction

Osteoarthritis is a common disease and a leading cause of disability worldwide (1), with pain being the primary reason that people seek medical care. In those with end stage disease, joint replacement surgery is a well-established, common and highly effective surgical procedure (2), where the majority of patients achieve substantial reductions in pain, improved joint function, mobility and health related quality of life (3). These operations cost the National Health Service (NHS) approximately £2 billion annually, which represents almost 1.5% of the entire NHS budget (4). Total knee replacements are a common surgery in the UK, with up to 100,000 performed each year (5) and with a lifetime risk of 10.8% for women and 8.1% for men undergoing knee replacement (6). The number of knee replacement surgeries are increasing in number due to an ageing and increasingly obese population (7,8) and are expected to continue to rise by 40% over the next 40 years (9,10).

Waiting times for NHS care have increased in recent years (11), whilst hospitals' capacity (the number of available theatres, surgeons and beds) is declining (7). NHS acute hospital trusts face challenges in planning hospital bed capacity, especially in winter months when patient demand for hospital care is very high. In the UK, prior to the COVID-19 pandemic, substantial challenges existed for delivering timely joint replacement surgery, with orthopaedics having the largest waiting list of any individual surgical specialty (12). Furthermore, planned operations are commonly cancelled, with 60% of cancellations happening on the day of surgery, usually due to lack of beds, staff, and/or operating capacity, which can be compounded during the winter months (13).

Patients remaining in hospital for longer than is necessary following surgery results in fewer hospital beds being available for the next scheduled patients. The time patients should expect to remain in hospital following total knee replacement surgery has decreased in recent years (from 3.7 days to 3.0 days between 2006 and 2016) (14), which suggests that hospital capacity and waiting lists could

For peer review only - http://bmjopen.bmj.com/site/about/guidelines.xhtml

have scope to improve. Furthermore, shorter length of hospital stay does not reflect compromised patient care (15) and is financially more sustainable.

Waiting lists could be reduced if hospitals are able to plan the timing of surgeries in order to optimise hospital efficiency. There is a need to identify predictors of hospital throughput, to better plan elective surgery, by understanding the characteristics of patients at greatest risk of staying in hospital longer than medically necessary.

The aim of this study was to identify risk factors associated with prolonged length of hospital stay and staying in hospital longer than medically necessary following primary knee replacement surgery using linked routinely collected data from an NHS Trust in South-West England.

For peer review only

For peer review only - http://bmjopen.bmj.com/site/about/guidelines.xhtml

Methods

Data sources

This is a longitudinal observational study and is reported in line with the STROBE reporting guidelines (16). Routinely collected data from an NHS Trust in England were used to identify patients receiving primary knee elective surgeries between 2016 and 2019. The Trust's electronic health records (EHR) were used to identify elective knee surgeries, using a combination of OPCS4 procedure and surgical site codes (see Supplementary Material Table S1). Further, the EHR were used to extract data for patient demographics (age, sex, deprivation quintile and comorbidities), and for admission details (length of stay and time and date of admission and discharge). Information describing the Trust's ratio of emergency to elective surgeries, daily emergency (non-elective) admissions and daily non-elective occupied beds were obtained from the Hospital Episode Statistics admitted patient care (HES-APC) dataset (17).

Outcome variables

A continuous measure of length of stay was generated to summarise the number of days between admission and discharge, up to 30 days; those in hospital for more than 30 days were coded as missing for this variable (this was to exclude outliers and excluded only 0.92% (N=21) of complete cases from analysis). Two binary outcome variables were derived, one indicating if patient's hospital stay was longer than 7 days (0= ≤7 days, 1= >7 days) and one indicating if patient had a recorded medically fit for discharge date that preceded their discharge date (0= did not have a medically fit for discharge date recorded, 1= patient remained in hospital when they were medically fit for discharge). Patients without a medically fit for discharge date were assumed to have been discharged when medically fit.

For peer review only - http://bmjopen.bmj.com/site/about/guidelines.xhtml

Predictive factors

Variables in the multivariable models include: a continuous measure of age on admission, patient sex (0= male, 1= female), patient area level deprivation (IMD quintile derived from LSOA (18)), comorbidities (as measured by the weighted Charlson Comorbidity Index (19)), a categorical measure of time since last discharge (0-2 months, 2-12 months, 12 months or more, or no previous admissions), a categorical measure of admission hour (06.00-12.00, 12.00-18.00, 18.00-06.00), the day of the week, season (winter: December-February; spring: March-May; summer: June-August; autumn: September-November) and year of admission, and the Trust monthly emergency/elective admissions ratio, daily non-elective admissions, and daily non-elective occupied beds.

Statistical analysis

Sample characteristics were summarised for all patients. To explore bias in data availability, descriptive statistics for key patient characteristics (age, sex, socioeconomic deprivation, ethnicity and comorbidities) were summarised for three groups: all patients, those with complete case data, and those who had a previous discharge. No notable differences in these characteristics were observed between these subsamples (see Table S2, Supplementary material). Complete case analysis was used throughout. The complete case sample was n=2295, or 76.3% of the whole sample.

Univariable and multivariable regression models were used to test associations between predictive factors and outcomes. Model assumptions were checked, and the following interactions were tested for: age and sex, age and comorbidities, age and season, age and deprivation, season and comorbidities, deprivation and comorbidities, season and deprivation. No interactions were observed. There was some evidence to suggest the continuous outcome (length of stay) violated the assumptions for the linear regression model (residuals not normally distributed), so robust standard errors were estimated using the Huber-White sandwich estimator (20).

For peer review only - http://bmjopen.bmj.com/site/about/guidelines.xhtml

Multivariable logistic regression models were used for binary outcomes and linear regression for the continuous outcome. The same 12 potential predictors (age, sex, area-level deprivation, comorbidities, days since last discharge, elective/non-elective ratio, non-elective admissions, non-elective occupied beds, admission hour and year, day and season of admission) were entered into the models for all outcomes. For each outcome, we ran least absolute shrinkage and selection operator (LASSO), elastic-net and ridge regression models. To produce the final models, we performed multivariable logistic (for binary outcomes)/linear (for continuous outcome) regression using backwards variable selection at p=0.1 and obtained C-statistics for the binary outcomes and R^2 for the continuous outcome. Calibration plots were produced for the final models for the binary outcomes.

Using the predictor variables retained in the backwards selection regression models, forest plots were produced to display the effects of each predictive factor for each outcome. All analyses were performed using Stata 17 (21).

Patient and public involvement

Patients and the public were consulted in a workshop for suggestions and comments to inform the development of the grant. They were again consulted in a follow-up workshop where initial results were presented. They were asked about their opinion on the prioritisation of particular patient groups (e.g. less complex patients during winter and complex patients in the summer) to maximise efficiency for surgery waiting lists.

Role of the funding source

The funders had no role in any of the following: the study design; the collection, analysis, and interpretation of data; the writing of the report; the decision to submit the paper for publication.

For peer review only - http://bmjopen.bmj.com/site/about/guidelines.xhtml

Results

Between January 2016 and December 2019, 3008 primary elective knee replacements were performed. Of these, 2295 (76.3%) patients had data available for all variables and were included in the complete case analysis (see Figure 1). As the continuous length of stay outcome measure was capped at 30 days, the multivariable analyses for this outcome included 2274 patients. Descriptive statistics are presented in Table 1 for the complete case sample. The mean length of stay for patients who were in hospital for 30 days or less was 5.0 days (SD = 3.9), 15.4% of patients stayed in hospital for more than 7 days. Most patients (92.9%) did not remain in hospital once they were medically fit for discharge, but 7.1% did have a recorded medically fit for discharge date which preceded their discharge date.

Multivariable backwards regression models are presented in Table 2. The full multivariable regression models, LASSO, elastic net and ridge regression models for each outcome are presented in Supplementary Tables S3-S5. The models for the binary outcomes (length of stay and medically fit for discharge) are accompanied by calibration plots (Figures S1-S4).

Remaining in hospital for more than seven days following surgery was associated with older age, higher deprivation, more comorbidities and being recently discharged from hospital (see Table 2/Figure 2). Associations between length of stay and time of admission were also observed but the confidence intervals were wide. Year and day of admission were also associated with length of the stay but without any apparent trend. No associations were found for sex, season of admission or hospital related factors (emergency over elective admissions ratio, non-elective admissions ratio or non-elective occupied beds).

Similarly, longer length of stay following surgery (measured using a continuous variable ranging from 0 to 30 days) was associated with older age, increasing deprivation, more comorbidities and recently being discharged from hospital (compared with never having been admitted to hospital or being

For peer review only - http://bmjopen.bmj.com/site/about/guidelines.xhtml

discharged from hospital at least over two months ago). Female sex was also associated with the continuous measure of length of stay. A spurious association with a wide confidence interval was again observed for admission hour. No associations were observed for hospital related factors, admission year, day or season.

Remaining in hospital when being medically fit for discharge was associated with older age, being female, greater deprivation, more comorbidities (though not the highest category of comorbidities, which was dropped from the model), being discharged from hospital recently (compared with 12 months or more or never), surgeries in 2017 and 2018 (compared with 2016). A weak association was observed with admission hour and no associations were found for hospital related factors, admission day or season.

The calibration plots (Figures 5-6) indicate close agreement for predicted and observed values for the binary length of stay and the medically fit for discharge outcome. The respective C-statistics of 0.72 and 0.74 also indicated good model discrimination.

Discussion

Summary of main findings

In this study, we observed that over 7% of patients remained in hospital beyond the date they were medically fit to be discharged, and 15% had long stays in hospital of more than 7 days. Being able to reduce this burden on hospital throughput would have a positive impact on overall hospital efficiency. Risk factors associated with remaining in hospital longer, and for longer than is necessary, included: older age, female patients with more comorbidities and those recently admitted to hospital. Patients from more deprived areas also had worse outcomes. There was no evidence of interactions with season, suggesting that efforts to limit operations to older patients and those with co-morbidities to summer months will not impact on hospital efficiency and throughput.

For peer review only - http://bmjopen.bmj.com/site/about/guidelines.xhtml

What is already known

Our results were in line with previous research showing longer length of stay is associated with older age, being female and worse health (14,22–24). Hospital and surgeon related factors (including hospital site, surgeon, time and day of surgery) (22) were also cited as predictors of length of stay, some of which are modifiable and presents opportunities to optimise efficiency, however, we found that hospital-related factors were not predictors of length of stay, although variables in this study, such as admission hour and day, were limited by small numbers in some categories. The factors associated with outcomes in our study were all patient related, including health-related and sociodemographic.

Strengths and limitations

This observational study had many strengths, including its reasonable sample size and use of robust statistical methods. This allowed us to identify a number of predictors with good discrimination and calibration. We also utilised three metrics of throughput as outcomes which demonstrated consistent findings. There are of course limitations to our study. There is great variation in practice and provision of services across the country (7) and, as a single centre study, the results may not be generalisable to other NHS trusts and settings. We only included data on operations performed in the NHS Trust and not privately funded patients. We purposefully analysed data from before the start of the COVID-19 pandemic, after which the healthcare landscape changed extraordinarily. Waiting times for knee replacement surgery have since increased, leading to huge burden on patients' wellbeing (25), and this is not represented in our results. There were missing data, however comparison of patient characteristics of those in full versus complete case dataset shows no evidence of selection (responder) bias (see Supplementary Table S2).

For peer review only - http://bmjopen.bmj.com/site/about/guidelines.xhtml

Implications for practice

Our models would allow health care providers to identify which patients are more likely to remain in hospital for longer, using these known variables, or risk factors. Whilst providers would not be able to change these risk factors, this knowledge could be used in planning surgeries and hospital capacity, potentially allowing the more complex surgeries with a longer predicted in-patient stay to be scheduled for when the hospital is under less strain, e.g., in the summer months.

Rescheduling patients' surgeries due to personal characteristics could be considered controversial, or potentially introducing inequalities. However, when asked if they would mind if surgeries were scheduled to a particular time based on patient characteristics, our PPI group said not, providing they were kept informed and could rely on the information being told to them. If there is evidence to support rescheduling patients in order to improve efficiency, patients and their caregivers could accept surgeries being moved.

We were able to build good-fitting models of length of stay and staying in hospital beyond being medically fit for discharge using data from all total knee replacements between 2016 and 2019 which can identify which patients are more likely to stay in hospital for longer and for longer than is necessary. These models may be used in planning bed capacity and potentially reduce waiting list times.

For peer review only

Contributors

RW, RM, MTR, and AJ drafted the original manuscript and all authors contributed to its conceptualisation, investigation, methods, validation, review, final approval, and accountability. RW and RM analysed the data. MTR and AJ supervised the project.

Declaration of interests

We declare no competing interests.

Funding

This study was funded by the Health Data Research (HDR) UK South West Better Care Partnership (#6.12). This research was supported by the National Institute for Health Research (NIHR) Applied Research Collaboration West (ARC West) at University Hospitals Bristol and Weston NHS Foundation Trust (core NIHR infrastructure funded: NIHR200181).

Data availability statement

Data may be obtained from a third party and are not publicly available. The data used in the study are collected by the North Bristol Trust (NBT) as part of their care and support. Sharing of anonymised data with the University of Bristol was underpinned by a data sharing agreement and solely covers the purposes of this study. Data requests can be made through the NBT.

For peer review only - http://bmjopen.bmj.com/site/about/guidelines.xhtml

References

1. Vos T, Flaxman AD, Naghavi M, Lozano R, Michaud C, Ezzati M, et al. Years lived with disability (YLDs) for 1160 sequelae of 289 diseases and injuries 1990–2010: a systematic analysis for the Global Burden of Disease Study 2010. Lancet. 2012 Dec 15;380(9859):2163–96.

2. National Institute for Health and Care Excellence. Osteoarthritis: care and management (CG177) [Internet]. Nice Guideline. 2014 [cited 2022 Jun 22]. Available from: https://www.nice.org.uk/guidance/cg177/chapter/Recommendations#referral-for-consideration-of-joint-surgery-2

3. Ethgen O, Bruyerè O, Richy F, Dardennes C, Reginster JY. Health-Related Quality of Life in Total Hip and Total Knee Arthroplasty: A Qualitative and Systematic Review of the Literature. J Bone Jt Surg. 2004;86(5):963–74.

4. National Institute for Health and Care Excellence. Joint replacement (primary): hip, knee and shoulder [Internet]. 2020. Available from: https://www.nice.org.uk/guidance/ng157/resources/joint-replacement-primary-hip-knee-and-shoulder-pdf-66141845322181

5. Price AJ, Alvand A, Troelsen A, Katz JN, Hooper G, Gray A, et al. Knee replacement. Vol. 392, The Lancet. Lancet Publishing Group; 2018. p. 1672–82.

6. Culliford DJ, Maskell J, Kiran A, Judge A, Javaid MK, Cooper C, et al. The lifetime risk of total hip and knee arthroplasty: results from the UK general practice research database. Osteoarthr Cartil. 2012 Jun 1;20(6):519–24.

7. Briggs T. A national review of adult elective orthopaedic services in England: Getting it right first time [Internet]. 2015 [cited 2022 May 17]. Available from: https://gettingitrightfirsttime.co.uk/wp-content/uploads/2017/06/GIRFT-National-Report-Mar15-Web.pdf

8. Culliford DJ, Maskell J, Beard DJ, Murray DW, Price AJ, Arden NK. Temporal trends in hip and knee replacement in the United Kingdom: 1991 to 2006. J Bone Jt Surg - Ser B [Internet]. 2010 Jan 1 [cited 2022 May 17];92(1):130–5. Available from: https://online.boneandjoint.org.uk/doi/abs/10.1302/0301-620X.92B1.22654

9. Culliford D, Maskell J, Judge A, Cooper C, Prieto-Alhambra D, Arden NK. Future projections of total hip and knee arthroplasty in the UK: Results from the UK Clinical Practice Research Datalink. Osteoarthr Cartil. 2015 Apr 1;23(4):594–600.

10. Matharu G, Culliford D, Blom A, Judge A. Projections for primary hip and knee replacement surgery up to the year 2060: an analysis based on data from The National Joint Registry for England, Wales, Northern Ireland and the Isle of Man. Ann R Coll Surg Engl [Internet]. 2022 Jun [cited 2022 Jun 22];104(6):443–8. Available from: https://pubmed.ncbi.nlm.nih.gov/34939832/

11. Anandaciva S. Hidden from the targets : how long are patients waiting for NHS care ? [Internet]. The King's Fund. 2018 [cited 2022 May 17]. Available from: https://www.kingsfund.org.uk/blog/2018/02/hidden-targets-patients-waiting-nhs-care

12. British Orthopaedic Association. A message for people waiting for joint replacement and other orthopaedic surgery [Internet]. 2020 [cited 2022 Jun 22]. Available from: https://www.boa.ac.uk/resources/a-message-for-people-waiting-for-joint-replacement-and-

For peer review only - http://bmjopen.bmj.com/site/about/guidelines.xhtml

other-orthopaedic-surgery.html

13. Royal College of Surgeons. Lack of NHS beds halted vital operations last year [Internet]. 2020 [cited 2022 Jun 22]. Available from: https://www.rcseng.ac.uk/news-and-events/media-centre/press-releases/rcs-survey-lack-of-nhs-beds-halted-operations/

14. Sarpong NO, Boddapati V, Herndon CL, Shah RP, Cooper HJ, Geller JA. Trends in Length of Stay and 30-Day Complications After Total Knee Arthroplasty: An Analysis From 2006 to 2016. J Arthroplasty [Internet]. 2019 Aug 1 [cited 2022 May 16];34(8):1575–80. Available from: https://www.sciencedirect.com/science/article/pii/S0883540319303596?casa_token=b9fcXF6p0ccAAAAA:L_PRg0vfPDLJGruYME1HOUHgZJTwj893jR_Osd-FaddD3ONJRdXitJOrUKfSU7SpzaG14Q4

15. Sutton JC, Antoniou J, Epure LM, Huk OL, Zukor DJ, Bergeron SG. Hospital discharge within 2 days following total hip or knee arthroplasty does not increase major-complication and readmission rates. J Bone Jt Surg - Am Vol [Internet]. 2016 [cited 2022 May 17];98(17):1419–28. Available from: https://journals.lww.com/jbjsjournal/Fulltext/2016/09070/Hospital_Discharge_within_2_Days_Following_Total.1.aspx

16. Vandenbroucke J, Von Elm E, Altman D, Gøtzsche P, Mulrow C, Pocock S. Strengthening the Reporting of Observational Studies in Epidemiology (STROBE): explanation and elaboration. Ann Intern Med. 2007;147(8):573–8.

17. Herbert A, Wijlaars L, Zylbersztejn A, Cromwell D, Hardelid P. Data Resource Profile: Hospital Episode Statistics Admitted Patient Care (HES APC). Int J Epidemiol [Internet]. 2017 Aug 1 [cited 2022 Aug 8];46(4):1093. Available from: /pmc/articles/PMC5837677/

18. Payne R, Abel G. UK indices of multiple deprivation - a way to make comparisons across constituent countries. London: Health Statistics Quarterly Office for National Statistics; 2012.

19. Charlson M, Szatrowski TP, Peterson J, Gold J. Validation of a combined comorbidity index. J Clin Epidemiol. 1994 Nov 1;47(11):1245–51.

20. Kirkwood BR, Sterne JA. Essential Medical Statistics. 2nd Editio. Blackwell Publishing Ltd; 2010.

21. StataCorp. Stata Statistical Software: Release 17. College Station, TX: StataCorp LLC; 2021.

22. Piuzzi NS, Strnad GJ, Ali Sakr Esa W, Barsoum WK, Bloomfield MR, Brooks PJ, et al. The Main Predictors of Length of Stay after Total Knee Arthroplasty: Patient-Related or Procedure-Related Risk Factors. J Bone Jt Surg - Am Vol [Internet]. 2019 Jun 19 [cited 2022 May 17];101(12):1093–101. Available from: https://journals.lww.com/jbjsjournal/Fulltext/2019/06190/The_Main_Predictors_of_Length_of_Stay_After_Total.7.aspx

23. Husted H, Holm G, Jacobsen S. Predictors of length of stay and patient satisfaction after hip and knee replacement surgery: Fast-track experience in 712 patients. Acta Orthop [Internet]. 2008;79(2):168–73. Available from: https://www.tandfonline.com/action/journalInformation?journalCode=iort20

24. Sephton BM, Bakhshayesh P, Edwards TC, Ali A, Kumar Singh V, Nathwani D. Predictors of extended length of stay after unicompartmental knee arthroplasty. J Clin Orthop Trauma. 2020 Mar 1;11:S239–45.

25. Clement ND, Scott CEH, Murray JRD, Howie CR, Deehan DJ, Farrow L, et al. The number of patients "worse than death" while waiting for a hip or knee arthroplasty has nearly doubled

For peer review only - http://bmjopen.bmj.com/site/about/guidelines.xhtml

during the COVID-19 pandemic. https://doi.org/101302/0301-620X103BBJJ-2021-0104R1 [Internet]. 2021 Mar 23 [cited 2022 May 17];103-B(4):672–80. Available from: https://online.boneandjoint.org.uk/doi/abs/10.1302/0301-620X.103B.BJJ-2021-0104.R1

For peer review only

For peer review only - http://bmjopen.bmj.com/site/about/guidelines.xhtml

Tables

Table 1: Characteristics of complete cases who have undergone NBT elective admissions for primary knee replacements (2015-2019) (N=2295)

Variable	N (%)[1]	Proportion (%) admitted >7 days	Mean length of stay (SD) N=2274	Proportion (%) medically fit for discharge
**Age at admission**				
0-34	19 (0.8)	5.3	3.1 (2.2)	0
35-44	54 (2.4)	7.4	3.4 (2.1)	0
45-54	231 (10.0)	7.8	3.8 (2.7)	1.7
55-64	519 (22.6)	70.2	4.3 (3.2) (N=517)	5.6
65-74	825 (36.0)	15.0	5.1 (3.9) (N=819)	6.1
75-84	551 (24.0)	20.2	5.7 (4.3) (N=543)	9.4
85+	96 (4.2)	43.8	8.0 (4.7) (N=91)	29.2
Age at admission, mean (SD)	67.4 (11.3)	-	-	-
**Sex**				
Female	1381 (60.2)	15.3	5.1 (3.9) (N=1367)	8.5
Male	914 (39.8)	15.5	4.7 (3.8) (N=907)	5.0
**Deprivation (IMD score)**				
1 (least deprived)	588 (25.6)	11.1	4.4 (3.1) (N=583)	
2	564 (24.6)	18.3	5.1 (4.0)	

For peer review only - http://bmjopen.bmj.com/site/about/guidelines.xhtml

			(N=555)	
3	395 (17.2)	17.0	5.2 (4.5)<br>(N=391)	
4	418 (18.2)	14.8	5.0 (3.7)<br>(N=416)	
5 (most deprived)	330 (14.4)	17.0	5.2 (4.2)<br>(N=329)	
**Ethnicity[2]**				
Non-White	76 (5.3)	-	-	-
Asian	25 (1.7)	-	-	-
Black	31 (2.2)	-	-	-
Mixed	10 (0.7)	-	-	-
Other	10 (0.7)	-	-	-
White	1368 (94.7)	-	-	-
Unknown	851	-	-	-
**Comorbidities (weighted Charlson index)**				
0	1260 (54.9)	12.0	4.5 (3.4)<br>(N=1255)	4.6
1-2	867 (37.8)	17.9	5.4 (4.1)<br>(N=854)	9.5
3-4	148 (6.5)	26.4	6.6 (5.3)<br>(N=145)	15.5
>=5	20 (0.9)	8.0	7.8 (5.5)	0
**Time since last discharge**				
0-2 months	338 (14.7)	26.9	6.3 (5.0)<br>(N=332)	
2-12 months	474 (20.7)	18.8	5.5 (4.2)	

For peer review only

For peer review only - http://bmjopen.bmj.com/site/about/guidelines.xhtml

			(N=470)	
12 months or more	409 (17.8)	13.0	4.6 (3.4)	
never	1074 (46.8)	11.1	4.4 (3.3)<br>(N=1063)	
**Emergency over elective admissions ratio (general/acute, monthly)**, Mean ± SD	0.8 ± 0.1	-	-	-
**Emergency admissions, daily**, Mean ± SD	152.6 ± 21.2	-	-	-
**Emergency occupied beds, daily**, Mean ± SD	914.9 ± 43.4	-	-	-
**Admission hour category**				
24.00-06.00	2 (0.1)	50.0	9.5 (3.5)	0
06.00-12.00	2092 (91.2)	14.4	4.9 (3.7)<br>(N=2075)	6.7
12.00-18.00	195 (8.5)	22.6	5.6 (4.7)<br>(N=192)	9.7
18.00-24.00	6 (0.3)	100.0	14.2 (7.0)<br>(N=5)	50.0
**Year of Admission**				
2016	185 (8.1)	10.8	4.7 (3.1)	2.7
2017	783 (34.1)	15.6	4.9 (3.6)<br>(N=770)	7.2
2018	629 (27.4)	18.4	5.3 (4.1)<br>(N=626)	9.1
2019	698 (30.4)	13.6	4.8 (4.1)<br>(N=693)	6.5

For peer review only - http://bmjopen.bmj.com/site/about/guidelines.xhtml

**Month of Admission**				
January	131 (5.7)	20.6	5.4 (4.7)	8.4
February	170 (7.4)	14.1	4.9 (4.3)	3.5
March	208 (9.1)	18.8	5.1 (4.2) (N=204)	7.2
April	183 (8.0)	17.5	4.9 (3.0) (N=181)	6.6
May	194 (8.5)	17.0	5.1 (3.8) (N=192)	9.3
June	194 (8.5)	9.8	4.4 (3.0) (N=192)	7.2
July	188 (8.2)	16.0	4.9 (4.1) (N=185)	10.6
August	164 (7.2)	15.2	4.9 (3.9) (N=161)	5.5
September	187 (8.2)	14.4	5.2 (4.1)	8.0
October	254 (11.1)	15.8	5.0 (4.0) (N=251)	5.9
November	260 (11.3)	13.5	4.9 (3.8) (N=258)	8.1
December	162 (7.1)	13.6	5.0 (3.5)	4.3
**Day of the Week of Admission**				
Sunday	2 (0.1)	50.0	8.5 (10.6)	50.0
Monday	495 (21.6)	18.4	4.8 (3.8) (N=490)	5.5
Tuesday	467 (20.4)	17.3	4.9 (3.7) (N=461)	8.8
Wednesday	403 (17.6)	13.2	5.0 (4.4)	7.7

For peer review only

			(N=399)	
Thursday	408 (17.8)	14.2	5.0 (3.5) (N=406)	6.1
Friday	467 (20.4)	13.9	5.1 (3.9) (N=463)	7.5
Saturday	53 (2.3)	7.6	4.7 (3.2) (N=53)	5.7
**Season of Admission**				
Winter (Dec-Feb)	463 (20.2)	15.8	5.1 (4.1)	5.2
Spring (Mar-May)	585 (25.5)	17.8	5.0 (3.7) (N=577)	7.7
Summer (Jun-Aug)	546 (23.8)	13.6	4.7 (3.7) (N=538)	7.9
Autumn (Sep-Nov)	701 (30.5)	14.6	5.0 (3.9) (N=696)	7.3
**Spell length of stay**, Mean ± SD	5.5 ± 6.1	-	-	-
**Spell length of stay up to 30 days**, Mean ± SD **(N=2274)**	5.0 ± 3.9	-	-	-
**Patients staying >7 days**				
<=7 (no)	1942 (84.6)	-	-	-
>7 (yes)	353 (15.4)	-	-	-
**Patients medically fit for discharge (MFFD)**				
with MFFD date before discharge	163 (7.1)	-	-	-
no MFFD date	2132 (92.9)	-	-	-

For peer review only - http://bmjopen.bmj.com/site/about/guidelines.xhtml

**Days between MFFD date and Discharge**, Mean ± SD (N=163)	7.2 ± 13.7	-	-	-

[1] n (%) for categorical, mean ± SD for continuous variables

[2] Percentages are for known data. Not included in analysis due to large number of ‘unknowns’

For peer review only

For peer review only - http://bmjopen.bmj.com/site/about/guidelines.xhtml

Table 2: Predictors of length of stay and remaining in hospital beyond being medically fit for discharge

Variable	Length of stay (admissions > 7 days) N=2295	Length of stay (≤ 30 days) N=2274[1]	Medically fit for discharge N=2275[2]
	OR (95% CI)	Coef (95% CI)	OR (95% CI)
**Age at admission**	1.06 (1.04 to 1.07)*	0.08 (0.07 to 0.09)*	1.07 (1.05 to 1.09)*
**Sex (female vs male)**	-	0.36 (0.06 to 0.67)*	1.71 (1.19 to 2.47)*
**Deprivation (IMD score)**			
1 (least deprived)	1.00	0.00	1.00
2	1.74 (1.22 to 2.47)*	0.65 (0.26 to 1.04)*	1.76 (1.05 to 2.96)*
3	1.74 (1.18 to 2.58)*	0.80 (0.32 to 1.28)*	1.84 (1.05 to 3.23)*
4	1.54 (1.04 to 2.28)*	0.75 (0.33 to 1.16)*	2.07 (1.20 to 3.57)*
5 (most deprived)	2.03 (1.34 to 3.05)*	0.98 (0.47 to 1.48)*	2.27 (1.27 to 4.06)*
**Comorbidities (weighted Charlson Comorbidities index)**			
0	1.00	0.00	1.00
1-2	1.40 (1.08 to 1.81)*	0.63 (0.31 to 0.94)*	1.89 (1.32 to 2.71)*
3-4	1.85 (1.20 to 2.86)*	1.42 (0.55 to 2.29)*	2.56 (1.48 to 4.42)*
≥5	3.03 (1.14 to 8.05)*	2.48 (0.15 to 4.81)*	-
**Time since Last Discharge**			
0-2 months	1.00	0.00	1.00
2-12 months	0.67 (0.47 to 0.95)*	-0.65 (-1.29 to -0.02)*	0.97 (0.60 to 1.57)
12 months or more	0.41 (0.27 to 0.61)*	-1.45 (-2.06 to -0.84)*	0.53 (0.30 to 0.92)*
Never	0.38 (0.28 to 0.53)*	-1.49 (-2.05 to -0.93)*	0.53 (0.33 to 0.84)*
**Emergency over elective admissions ratio**	-	-	-
**NEL admissions**	-	-	-

For peer review only - http://bmjopen.bmj.com/site/about/guidelines.xhtml

**NEL occupied beds**	-	-	-
**Admission hour category**			
06.00-12.00	1.00	0.00	1.00
12.00-18.00	1.81 (1.23 to 2.66)*	0.79 (0.17 to 1.41)*	1.56 (0.91 to 2.66)
18.00-06:00	77.21 (8.01 to 744.20)*	7.55 (2.85 to 12.24)*	8.11 (1.74 to 37.75)*
**Year of Admission**			
2016	1.00	0.00	1.00
2017	1.66 (0.96 to 2.86)	0.20 (-0.27 to 0.67)	2.95 (1.14 to 7.64)*
2018	1.96 (1.12 to 3.42)*	0.47 (-0.04 to 0.99)	3.89 (1.49 to 10.12)*
2019	1.14 (0.65 to 1.99)	-0.21 (-0.71 to 0.30)	2.32 (0.88 to 6.09)
**Day of Admission**			
Sunday	-	-	-
Monday	1.00	-	-
Tuesday	0.89 (0.62 to 1.26)	-	-
Wednesday	0.59 (0.40 to 0.88)*	-	-
Thursday	0.66 (0.45 to 0.97)*	-	-
Friday	0.78 (0.54 to 1.13)	-	-
Saturday	0.20 (0.06 to 0.73)*	-	-
**Season of Admission**			
Winter (Dec-Feb)	1.00	-	-
Spring (Mar-May)	1.14 (0.80 to 1.61)	-	-
Summer (Jun-Aug)	0.73 (0.50 to 1.06)	-	-
Autumn (Sep-Nov)	0.90 (0.64 to 1.28)	-	-
	**C-statistic (95% CI)**	**R^2**	**C-statistic (95% CI)**
	0.72 (0.69 to 0.74)	0.130	0.74 (0.71 to 0.78)

[1] included sample N=2274 patients with length of stay ≤30 days

[2] included sample N=2295 minus those excluded when CCI score ≥5 was dropped from the model (N=20)

For peer review only - http://bmjopen.bmj.com/site/about/guidelines.xhtml

Figures

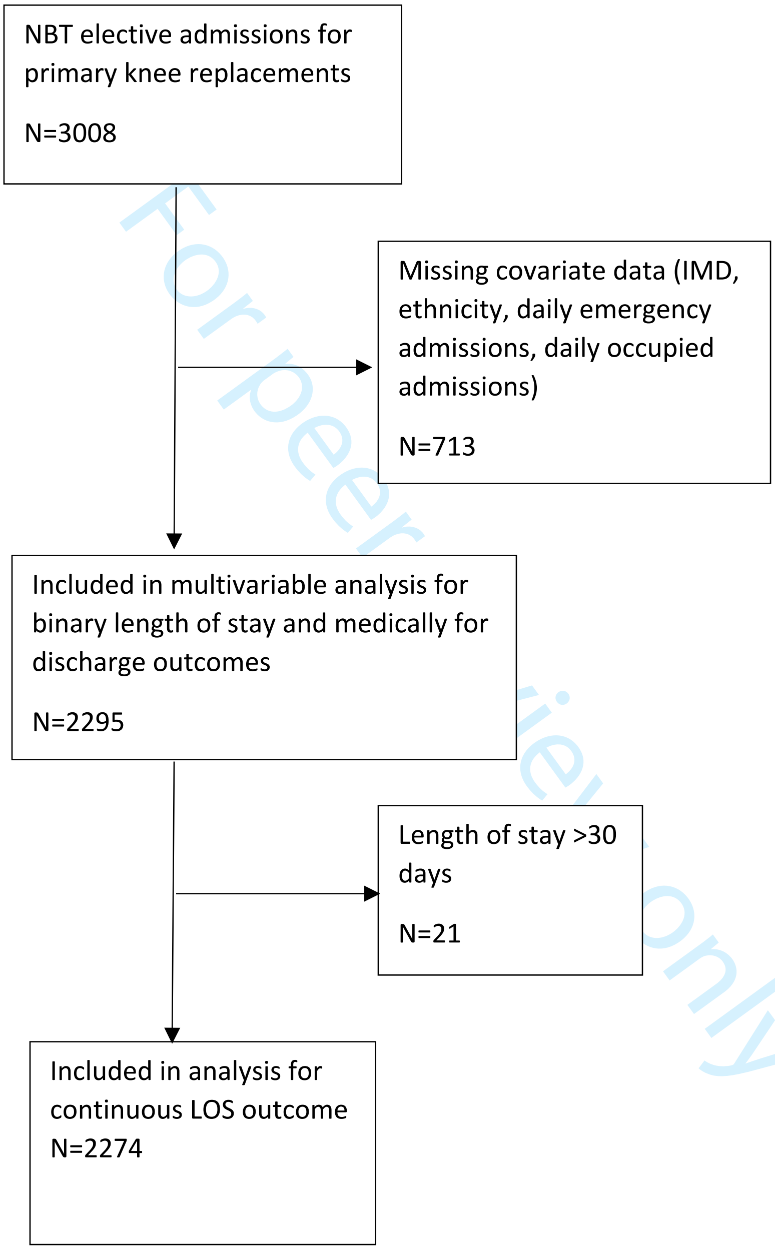

Figure 1: Flow diagram of patients excluded and included in the study

For peer review only

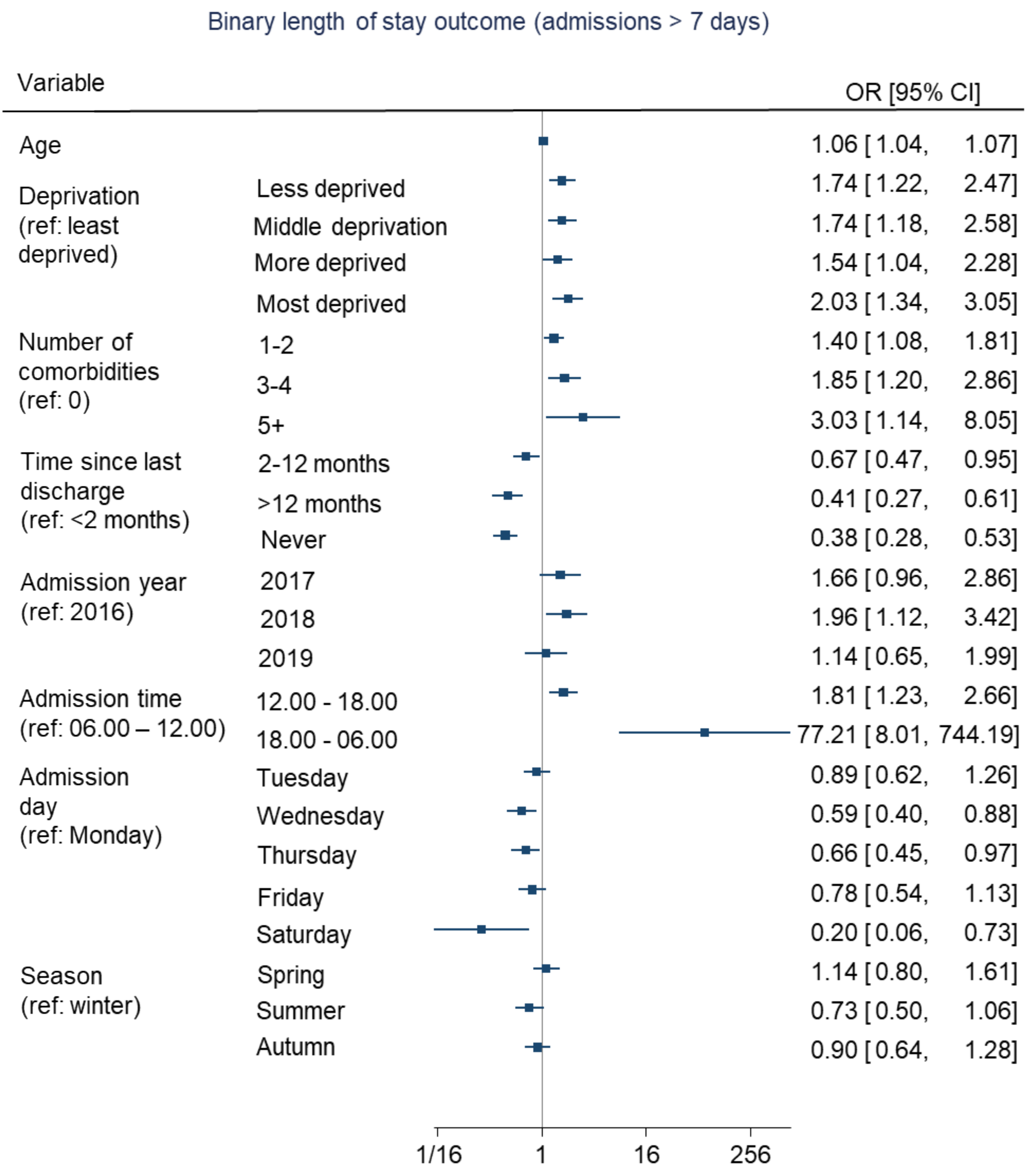

Figure 2: Predictors for length of stay of more than seven days for patients who had undergone knee replacement surgery

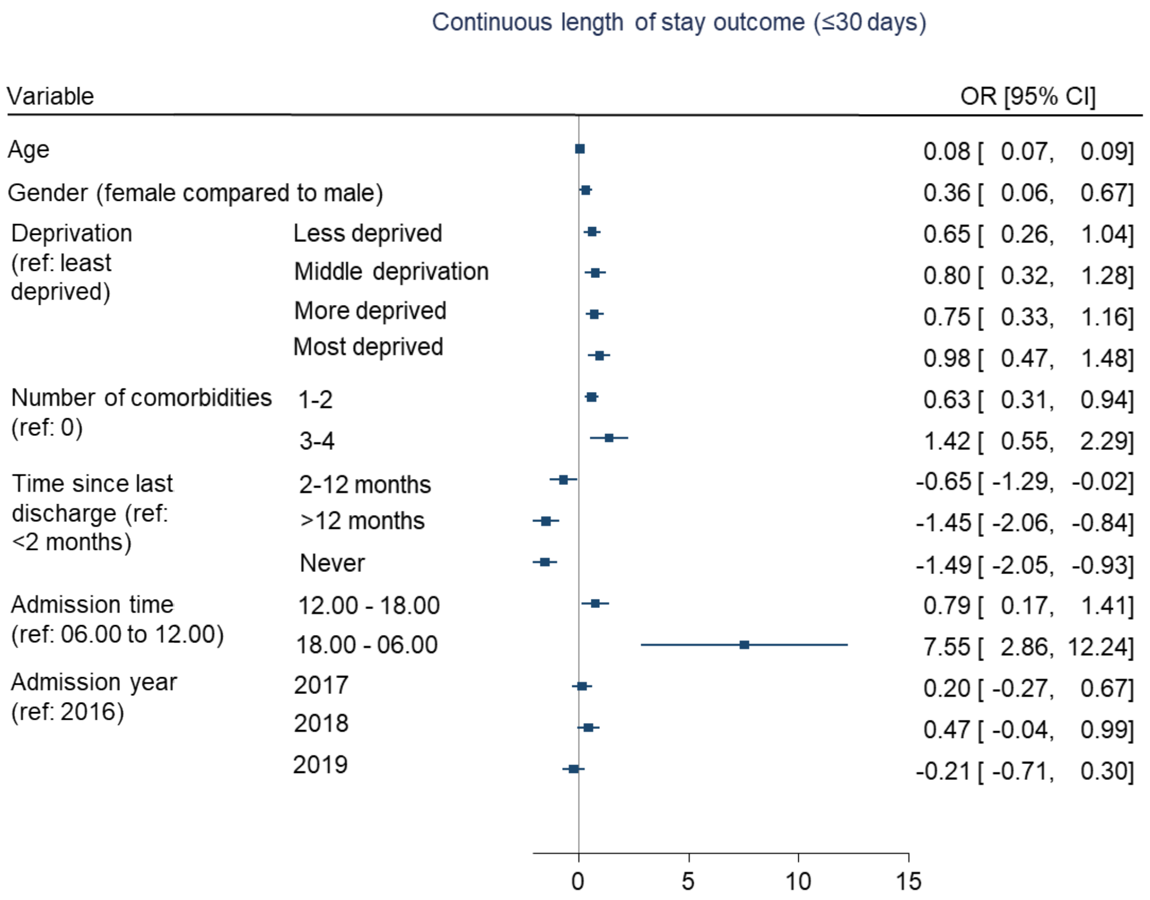

Figure 3: Predictors of length of stay (using a continuous measure) in patients following knee replacement surgery

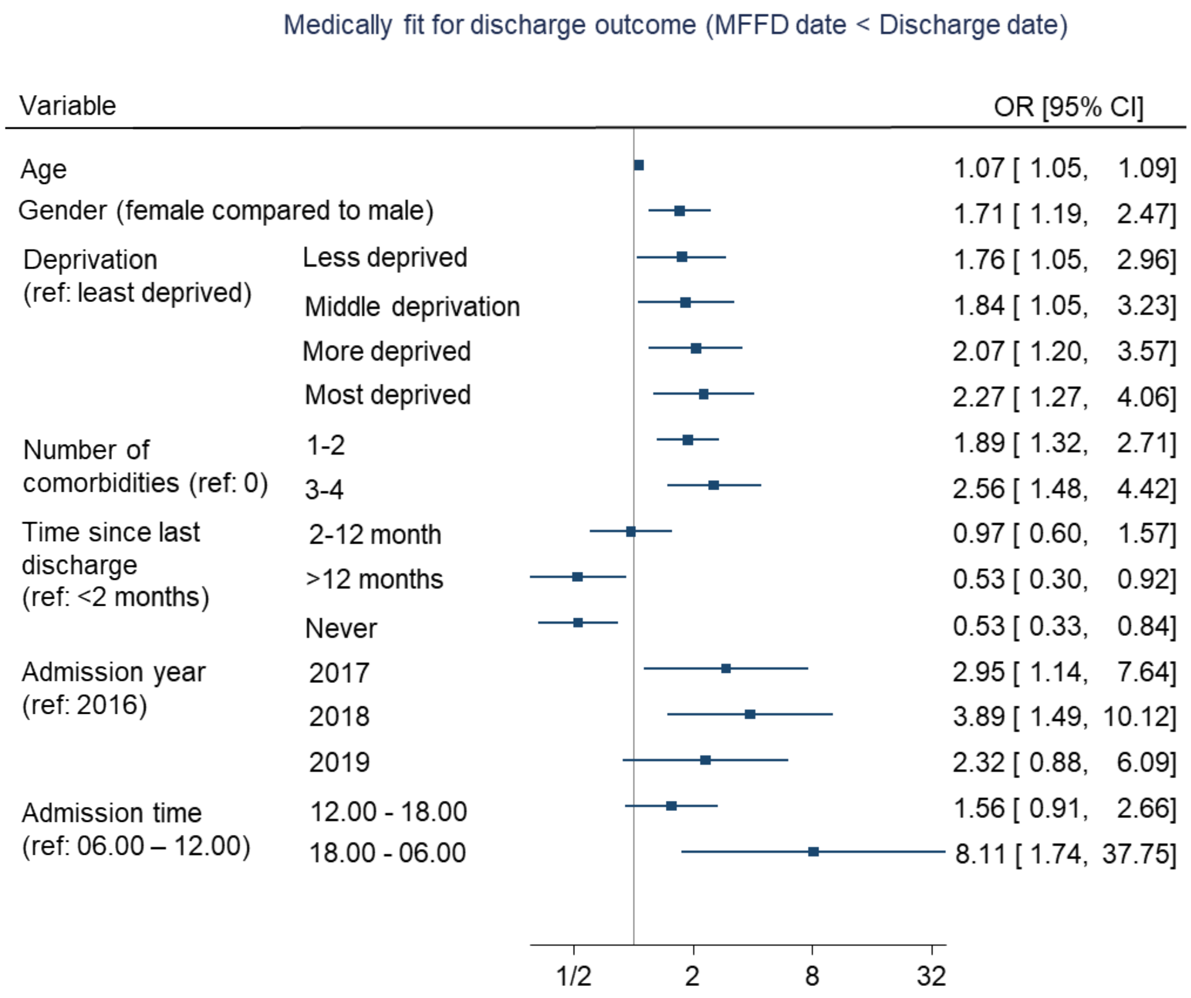

Figure 4: Predictors of remaining in hospital beyond being declared medically fit after knee replacement surgery

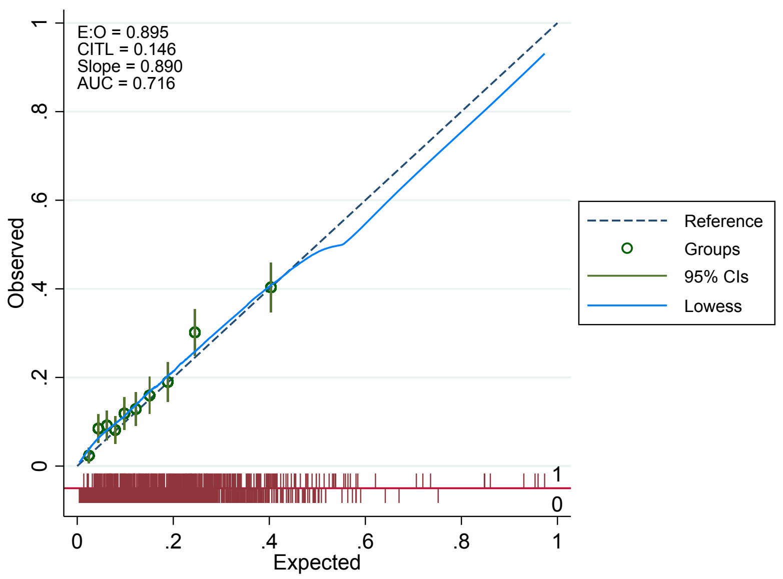

Figure 5: Calibration plot for final model for binary length of stay outcome

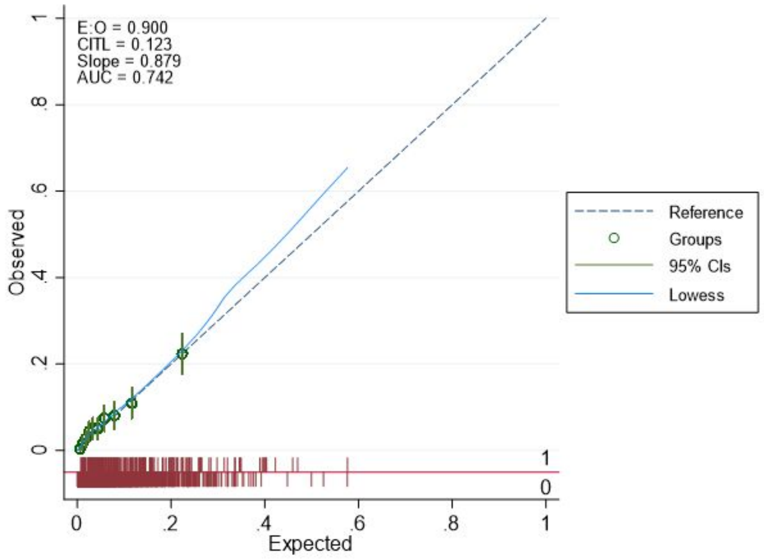

Figure 6: Calibration plot for final model for medically fit for discharge outcome

Supplementary material

Supplementary Table S1: OPCS-4 codes used to identify primary hip and knee replacement operations

Supplementary Table S2: Sample characteristics for total sample (N=3008), those with complete data and with a previous admission (N=1221) and those with complete data and no previous admission (N=2295)

Supplementary Table S3: Multivariable models for binary length of stay outcome (admissions > 7 days)

Supplementary Figure S1: Calibration plot for binary length of stay outcome (full regression model)

Supplementary Figure S2: Calibration plot for binary length of stay outcome (backwards selection regression model)

Supplementary Table S4: Multivariable models for continuous length of stay outcome (≤ 30 days)

Supplementary Table S5: Multivariable models for medically fit for discharge date outcome (MFFD date < discharge date)

Supplementary Figure S3: Calibration plot for medically fit for discharge (full regression model)

Supplementary Figure S4: Calibration plot for medically fit for discharge (backwards selection regression model)

For peer review only

Supplementary Table S1: OPCS-4 codes used to identify primary hip and knee replacement operations

Category	Code	Description	Notes
Primary Total Knee Replacement	W40.1	Primary total prosthetic replacement of knee joint using cement	
	W40.8	Other specified total prosthetic replacement of knee joint using cement	
	W40.9	Unspecified total prosthetic replacement of knee joint using cement	
	W41.1	Primary total prosthetic replacement of knee joint not using cement	
	W41.8	Other specified total prosthetic replacement of knee joint not using cement	
	W41.9	Unspecified total prosthetic replacement of knee joint not using cement	
	W42.1	Primary total prosthetic replacement of knee joint NEC	
	W42.8	Other specified other total prosthetic replacement of knee joint	
	W42.9	Unspecified other total prosthetic replacement of knee joint	
	O18.1	Primary hybrid prosthetic replacement of knee joint using cement	
	O18.8	Other specified hybrid prosthetic replacement of knee joint using cement	
	O18.9	Unspecified hybrid prosthetic replacement of knee joint using cement	
Resurfacing / Reconstruction	W58.1	Primary resurfacing arthroplasty of joint	Require combination with site + combination codes to ID
	W58.8	Other specified reconstruction of joint	Require combination with site + combination codes to ID
	W58.9	Unspecified other reconstruction of joint	Require combination with site + combination codes to ID
Primary unicondylar / unicompartmental knee operations	W52.1	Primary prosthetic replacement of articulation of bone using cement NEC	Require combination with site + combination codes to ID
	W52.8	Other specified prosthetic replacement of articulation of other bone using cement	Require combination with site + combination codes to ID

For peer review only

W52.9	Unspecified prosthetic replacement of articulation of other bone using cement	Require combination with site + combination codes to ID
W53.1	Primary prosthetic replacement of articulation of bone not using cement NEC	Require combination with site + combination codes to ID
W53.9	Unspecified prosthetic replacement of articulation of other bone not using cement	Require combination with site + combination codes to ID
W54.0	Conversion from previous prosthetic replacement of articulation of bone NEC	Require combination with site + combination codes to ID
W54.1	Primary prosthetic replacement of articulation of bone NEC	Require combination with site + combination codes to ID
W54.8	Other specified other prosthetic replacement of articulation of other bone	Require combination with site + combination codes to ID
W54.9	Unspecified other prosthetic replacement of articulation of other bone	Require combination with site + combination codes to ID

For peer review only

Supplementary Table S2: Sample characteristics for total sample (N=3008), those with complete data and with a previous admission (N=1221) and those with complete data and no previous admission (N=2295)

Main characteristics		Total sample N=3008	Complete case sample included in multivariable model with continuous days since last discharge variable N=1221	Complete case sample included in multivariable model with categorical days since last discharge variable N=2295
Age, mean (SD)		67.5 (11.4)	68.0 (11.2)	67.4 (11.3)
Sex, N (%)	Female	1803 (59.9)	734 (60.1)	1381 (60.2)
	Male	1205 (40.1)	487 (39.9)	914 (39.8)
IMD score, N (%)	1 (least deprived)	753 (25.0)	310 (25.4)	588 (25.6)
	2	719 (23.9)	297 (24.3)	564 (24.6)
	3	515 (17.1)	225 (18.4)	395 (17.2)
	4	538 (17.9)	212 (17.4)	418 (18.2)
	5 (most deprived)	426 (14.2)	177 (14.5)	330 (14.4)
	(missing)	57 (1.9)	-	-
Ethnicity, N (%)	Non-White	90 (3.0)	47 (3.9)	76 (3.3)
	White	1827 (60.7)	779 (63.8)	1368 (59.6)
	(missing)	1091 (36.3)	395 (32.4)	851 (37.1)
Comorbidities, N (%)	0	1681 (55.9)	618 (50.6)	1260 (54.9)
	1-2	1126 (37.4)	485 (39.7)	867 (37.8)
	3-4	175 (5.8)	101 (8.3)	148 (6.5)
	>=5	26 (0.9)	17 (1.4)	20 (0.9)

For peer review only

Supplementary Table S3: Multivariable models for binary length of stay outcome (admissions > 7 days) (N=2295)

**Variable**	**Multivariable logistic regression (no variable selection)**		**Lasso regression**	**Elastic net logistic regression**	**Ridge regression**	**Multivariable logistic regression (backwards selection)**	
	**OR (95%CI)**	**p**	**OR**	**OR**	**OR**	**OR (95%CI)**	**p**
**Age at admission**	1.06 (1.04 to 1.07)	<0.001	1.78	1.77	1.70	1.06 (1.04 to 1.07)	<0.001
**Sex (female vs male)**	0.91 (0.71 to 1.17)	0.470	0.98	0.98	0.97	-	
**Deprivation (IMD score)**							
1 (least deprived)	*1.00*		*1.00*	*1.00*	*1.00*	*1.00*	
2	1.74 (1.22 to 2.48)	0.002	1.20	1.20	1.19	1.74 (1.22 to 2.47)	0.002
3	1.76 (1.19 to 2.60)	0.005	1.17	1.16	1.16	1.74 (1.18 to 2.58)	0.005
4	1.55 (1.04 to 2.29)	0.030	1.12	1.12	1.12	1.54 (1.04 to 2.28)	0.030
5 (most deprived)	2.05 (1.36 to 3.09)	0.001	1.21	1.21	1.20	2.03 (1.34 to 3.05)	0.001
**Comorbidities (weighted Charlson index)**							
0	*1.00*		*1.00*	*1.00*	*1.00*	*1.00*	
1-2	1.40 (1.08 to 1.81)	0.011	1.15	1.15	1.16	1.40 (1.08 to 1.81)	0.011
3-4	1.86 (1.20 to 2.87)	0.005	1.15	1.15	1.16	1.85 (1.20 to 2.86)	0.006
>=5	2.96 (1.11 to 7.90)	0.030	1.10	1.10	1.11	3.03 (1.14 to 8.05)	0.026
**Time since Last Discharge**							

For peer review only

0-2 months	*1.00*		*1.00*	*1.00*	*1.00*	*1.00*	
2-12 months	0.66 (0.47 to 0.94)	0.023	0.89	0.89	0.91	0.67 (0.47 to 0.95)	0.025
12 months or more	0.40 (0.27 to 0.60)	<0.001	0.75	0.75	0.77	0.41 (0.27 to 0.61)	<0.001
never	0.38 (0.27 to 0.53)	0.761	0.65	0.66	0.68	0.38 (0.28 to 0.53)	<0.001
**Emergency over elective admissions ratio**	0.65 (0.06 to 7.72)	0.736	-	-	0.98	-	
**NEL admissions**	1.00 (0.99 to 1.01)	0.729	-	-	1.03	-	
**NEL occupied beds**	0.999 (0.995 to 1.004)	0.761	1.01	1.01	1.03	-	
**Admission hour category**							
06.00-12.00	*1.00*		*1.00*	*1.00*	*1.00*	*1.00*	
12.00-18.00	1.82 (1.23 to 2.68)	0.003	1.17	1.17	1.16	1.81 (1.23 to 2.66)	0.003
18.00-06:00	76.72 (7.91 to 744.34)	<0.001	1.26	1.26	1.25	77.21 (8.01 to 744.20)	<0.001
**Year of Admission**							
2016	*1.00*		*1.00*	*1.00*	*1.00*	*1.00*	
2017	1.70 (0.92 to 3.17)	0.093	1.16	1.16	1.12	1.66 (0.96 to 2.86)	0.071
2018	2.07 (1.05 to 4.08)	0.036	1.24	1.24	1.19	1.96 (1.12 to 3.42)	0.018
2019	1.21 (0.54 to 2.70)	0.639	-	1.00	0.94	1.14 (0.65 to 1.99)	0.658
**Day of the Week of Admission**							
Sunday							
Monday	*1.00*		*1.00*	*1.00*	*1.00*	*1.00*	

For peer review only

Tuesday	0.88 (0.62 to 1.25)	0.479	-	1.00	0.98	0.89 (0.62 to 1.26)	0.497
Wednesday	0.59 (0.39 to 0.87)	0.008	0.87	0.87	0.86	0.59 (0.40 to 0.88)	0.009
Thursday	0.65 (0.44 to 0.96)	0.031	0.91	0.90	0.89	0.66 0.45 to 0.97)	0.033
Friday	0.76 (0.52 to 1.12)	0.164	0.95	0.95	0.93	0.78 (0.54 to 1.13)	0.185
Saturday	0.21 (0.05 to 0.79)	0.021	0.84	0.84	0.85	0.20 (0.06 to 0.73)	0.014
**Season of Admission**							
Winter (Dec-Feb)	*1.00*		*1.00*	*1.00*	*1.00*	*1.00*	
Spring (Mar-May)	1.11 (0.77 to 1.62)	0.571	1.07	1.07	1.08	1.14 (0.80 to 1.61)	0.479
Summer (Jun-Aug)	0.70 (0.45 to 1.10)	0.121	0.91	0.91	0.91	0.73 (0.50 to 1.06)	0.102
Autumn (Sep-Nov)	0.87 (0.59 to 1.29)	0.490	0.98	0.98	0.96	0.90 (0.64 to 1.28)	0.562
C-statistic	0.73 (0.71 to 0.76)					0.72 (0.69 to 0.74)	

For peer review only

Fig S1: Calibration plot for length of stay (full regression model)

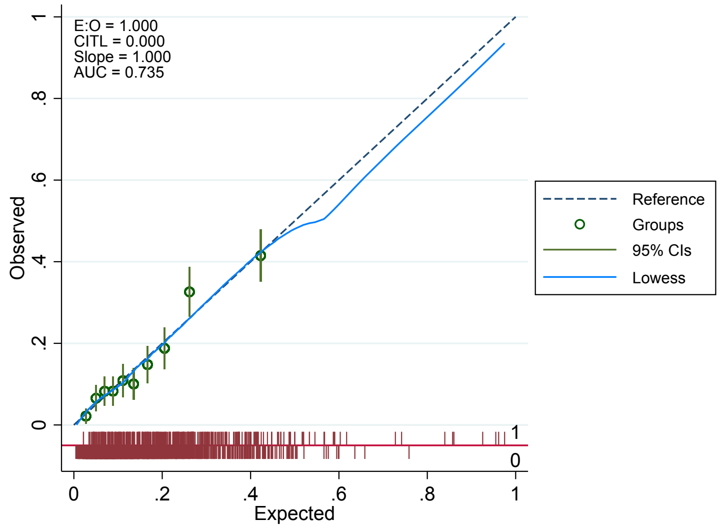

Fig S2: Calibration plot for length of stay (backwards selection regression model)

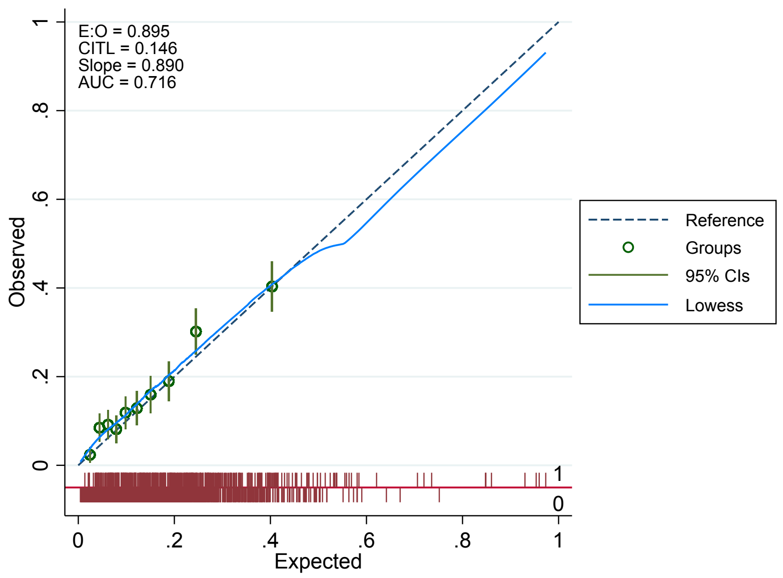

Supplementary Table S4: Multivariable models for continuous length of stay outcome (≤ 30 days) (N=2274)

Variable	Multivariable linear regression (no variable selection)		Lasso regression	Elastic net logistic regression	Ridge regression	Multivariable linear regression (backwards selection)	
	Coef (95%CI)	p	Coef	Coef	Coef	Coef (95%CI)	p
**Age at admission**	0.08 (0.07 to 0.09)	<0.001	0.87	0.87	0.83	0.08 (0.07 to 0.09)	<0.001
**Sex (female vs male)**	0.36 (0.05 to 0.66)	0.021	0.15	0.15	0.17	0.36 (0.06 to 0.67)	0.018
**Deprivation (IMD score)**							
1 (least deprived)	*0.00*		*0.00*	*0.00*	*0.00*	*0.00*	
2	0.62 (0.22 to 1.01)	0.002	0.18	0.18	0.20	0.65 (0.26 to 1.04)	0.001
3	0.81 (0.32 to 1.29)	0.001	0.22	0.22	0.24	0.80 (0.32 to 1.28)	0.001
4	0.73 (0.32 to 1.15)	0.001	0.20	0.20	0.22	0.75 (0.33 to 1.16)	<0.001
5 (most deprived)	0.96 (0.46 to 1.47)	<0.001	0.26	0.26	0.27	0.98 (0.47 to 1.48)	<0.001
**Comorbidities (weighted Charlson index)**							
0	*0.00*		*0.00*	*0.00*	*0.00*	*0.00*	
1-2	0.62 (0.31 to 0.93)	<0.001	0.27	0.27	0.28	0.63 (0.31 to 0.94)	<0.001
3-4	1.36 (0.50 to 2.23)	0.002	0.32	0.32	0.32	1.42 (0.55 to 2.29)	0.001
>=5	2.44 (0.12 to 4.75)	0.039	0.20	0.20	0.22	2.48 (0.15 to 4.81)	0.037
**Time since Last Discharge**							

For peer review only

0-2 months	*0.00*		*0.00*	*0.00*	*0.00*	*0.00*	
2-12 months	-0.65 (-1.29 to -0.01)	0.046	-0.15	-0.15	-0.13	-0.65 (-1.29 to -0.02)	0.044
12 months or more	-1.48 (-2.09 to -0.87)	<0.001	-0.46	-0.46	-0.42	-1.45 (-2.06 to -0.84)	<0.001
never	-1.50 (-2.06 to -0.94)	<0.001	-0.62	-0.62	-0.57	-1.49 (-2.05 to -0.93)	<0.001
**Emergency over elective admissions ratio**	-0.19 (-3.34 to 2.95)	0.904	-	-	-0.001	-	
**NEL admissions**	-0.01 (-0.02 to 0.01)	0.732	-	-	-0.05	-	
**NEL occupied beds**	-0.0005 (-0.0066 to 0.0057)	0.884	-	-	0.03	-	
**Admission hour category**							
06.00-12.00	*0.00*		*0.00*	*0.00*	*0.00*	*0.00*	
12.00-18.00	0.87 (0.24 to 1.50)	0.007	0.20	0.20	0.21	0.79 (0.17 to 1.41)	0.012
18.00-06:00	7.61 (2.91 to 12.31)	0.002	0.40	0.40	0.40	7.55 (2.85 to 12.24)	0.002
**Year of Admission**							
2016	*0.00*		*0.00*	*0.00*	*0.00*	*0.00*	
2017	0.36 (-0.30 to 1.03)	0.282	0.05	0.05	0.07	0.20 (-0.27 to 0.67)	0.411
2018	0.73 (0.04 to 1.41)	0.037	0.17	0.17	0.21	0.47 (-0.04 to 0.99)	0.073
2019	0.07 (-0.82 to 0.96)	0.879	-0.10	-0.10	-0.08	-0.21 (-0.71 to 0.30)	0.425
**Day of the Week of Admission**							

For peer review only

Sunday	-	-					
Monday	*0.00*		*0.00*	*0.00*	*0.00*		
Tuesday	1.11 (-0.34 to 0.56)	0.637	-	-	0.02		
Wednesday	0.13 (-0.37 to 0.64)	0.604	-	-	0.03		
Thursday	0.20 (-0.26 to 0.66)	0.399	0.02	0.02	0.06		
Friday	0.53 (0.05 to 1.01)	0.032	0.14	0.14	0.18		
Saturday	-0.49 (-1.55 to - 0.57)	0.365	-0.04	-0.04	-0.06		
**Season of Admission**							
Winter (Dec-Feb)	*0.00*		*0.00*	*0.00*	*0.00*		
Spring (Mar-May)	-0.13 (-0.62 to 0.36)	0.604	-	-	-0.02		
Summer (Jun-Aug)	-0.52 (-1.06 to 0.02)	0.060	-0.14	-0.14	-0.16		
Autumn (Sep-Nov)	-0.10 (-0.60 to 0.40)	0.700	-	-	-0.02		
R^2	0.136					0.130	

For peer review only

For peer review only - http://bmjopen.bmj.com/site/about/guidelines.xhtml

Supplementary Table S5: Multivariable models for binary medically fit for discharge date outcome (MFFD date < discharge date) (N=2275)

Variable	Multivariable logistic regression (no variable selection)		Lasso regression	Elastic net logistic regression	Ridge regression	Multivariable logistic regression (backwards selection)	
	**OR (95%CI)**	**p**	**OR**	**OR**	**OR**	**OR (95%CI)**	**p**
**Age at admission**	1.07 (1.05 to 1.09)	<0.001	2.13	2.11	1.95	1.07 (1.05 to 1.09)	<0.001
**Sex (female vs male)**	1.70 (1.17 to 2.45)	0.005	1.28	1.29	1.28	1.71 (1.19 to 2.47)	0.004
**Deprivation (IMD score)**							
1 (least deprived)	*1.00*		*1.00*	*1.00*	*1.00*	*1.00*	
2	1.78 (1.06 to 3.02)	0.031	1.22	1.24	1.19	1.76 (1.05 to 2.96)	0.033
3	1.89 (1.08 to 3.33)	0.026	1.22	1.23	1.18	1.84 (1.05 to 3.23)	0.032
4	2.12 (1.22 to 3.67)	0.007	1.28	1.29	1.24	2.07 (1.20 to 3.57)	0.009
5 (most deprived)	2.23 (1.24 to 4.01)	0.007	1.27	1.28	1.22	2.27 (1.27 to 4.06)	0.006
**Comorbidities (weighted Charlson index)**							
0	*1.00*		*1.00*	*1.00*	*1.00*	*1.00*	
1-2	1.91 (1.32 to 2.74)	0.001	1.35	1.35	1.33	1.89 (1.32 to 2.71)	0.001
3-4	2.40 (1.37 to 4.22)	0.002	1.24	1.25	1.25	2.56 (1.48 to 4.42)	0.001
>=5	-		0.79	0.77	0.83	-	
**Time since Last Discharge**							
0-2 months	*1.00*		*1.00*	*1.00*	*1.00*	*1.00*	

For peer review only

2-12 months	0.94 (0.58 to 1.52)	0.792	-	1.00	1.01	0.97 (0.60 to 1.57)	0.907
12 months or more	0.48 (0.27 to 0.85)	0.012	0.79	0.79	0.82	0.53 (0.30 to 0.92)	0.024
never	0.51 (0.32 to 0.82)	0.005	0.74	0.74	0.76	0.53 (0.33 to 0.84)	0.007
**Emergency over elective admissions ratio**	0.97 (0.02 to 37.81)	0.986	-	-	1.03	-	
**NEL admissions**	0.99 (0.98 to 1.01)	0.230	0.91	0.85	0.87	-	
**NEL occupied beds**	1.00 (1.00 to 1.01)	0.592	1.05	0.07	1.09	-	
**Admission hour category**							
06.00-12.00	*1.00*		*1.00*	*1.00*	*1.00*	*1.00*	
12.00-18.00	1.61 (0.92 to 2.81)	0.096	1.13	1.15	1.14	1.56 (0.91 to 2.66)	0.103
18.00-06:00	8.81 (1.77 to 43.87)	0.008	1.14	1.14	1.14	8.11 (1.74 to 37.75)	0.008
**Year of Admission**							
2016	*1.00*		*1.00*	*1.00*	*1.00*	*1.00*	
2017	2.91 (1.00 to 8.41)	0.049	1.37	1.41	1.20	2.95 (1.14 to 7.64)	0.026
2018	4.37 (1.43 to 13.37)	0.010	1.57	1.64	1.36	3.89 (1.49 to 10.12)	0.005
	2.78 (0.79 to 9.82)	0.112	1.26	1.36	1.11	2.32 (0.88 to 6.09)	0.088
**Day of the Week of Admission**							
Sunday							
Monday	*1.00*		*1.00*	*1.00*	*1.00*		

For peer review only

Tuesday	1.67 (0.99 to 2.82)	0.057	1.19	1.19	1.16		
Wednesday	1.32 (0.75 to 2.32)	0.337	1.08	1.08	1.07		
Thursday	1.04 (0.57 to 1.88)	0.901	-	-	0.99		
Friday	1.68 (0.96 to 2.93)	0.069	1.18	1.19	1.17		
Saturday	0.65 (0.16 to 2.68)	0.548	0.96	0.94	0.95		
**Season of Admission**							
Winter (Dec-Feb)	*1.00*		*1.00*	*1.00*	*1.00*		
Spring (Mar-May)	1.37 (0.78 to 2.44)	0.276	1.11	1.12	1.13		
Summer (Jun-Aug)	1.38 (0.72 to 2.65)	0.327	1.10	1.12	1.13		
Autumn (Sep-Nov)	1.54 (0.85 to 2.76)	0.152	1.14	1.17	1.15		
C-statistic	0.77 (0.74 to 0.81)					0.74 (0.71 to 0.78)	

For peer review only

Fig S3: Calibration plot medically fit for discharge (full regression model)

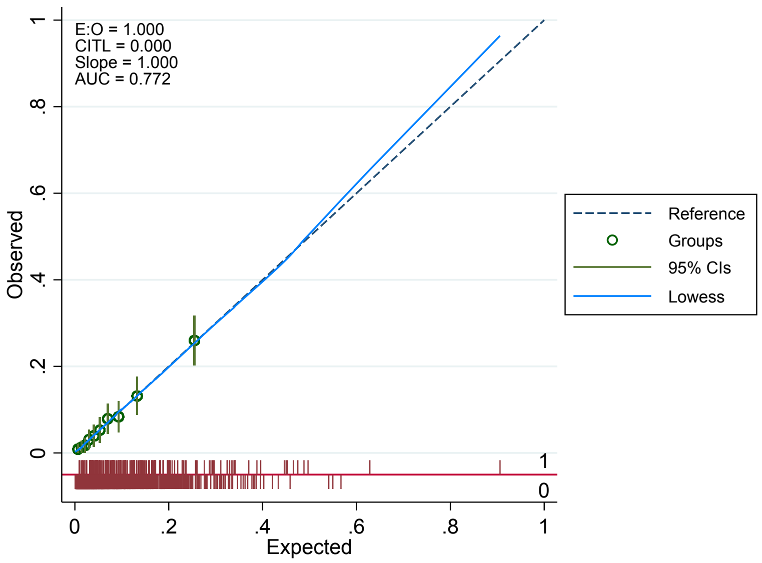

Fig S4: Calibration plot medically fit for discharge (backwards selection model)

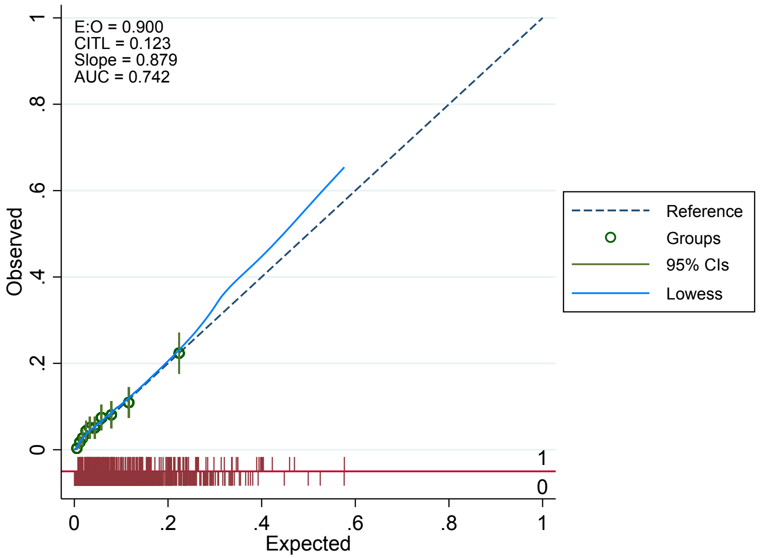

STROBE Statement—checklist of items that should be included in reports of observational studies

	Item No.	Recommendation	Page No.	Relevant text from manuscript
**Title and abstract**	1	(*a*) Indicate the study's design with a commonly used term in the title or the abstract	1	
		(*b*) Provide in the abstract an informative and balanced summary of what was done and what was found	1	
**Introduction**				
Background/rationale	2	Explain the scientific background and rationale for the investigation being reported	5-6	
Objectives	3	State specific objectives, including any prespecified hypotheses	6	"The aim of this study was to identify risk factors associated with prolonged length of hospital stay and staying in hospital longer than medically necessary following primary knee replacement surgery using linked routinely collected data from an NHS Trust in South-West England."
**Methods**				
Study design	4	Present key elements of study design early in the paper	7	"This is a longitudinal observational study"
Setting	5	Describe the setting, locations, and relevant dates, including periods of recruitment, exposure, follow-up, and data collection	7	Data sources section
Participants	6	(*a*) *Cohort study*—Give the eligibility criteria, and the sources and methods of selection of participants. Describe methods of follow-up<br>*Case-control study*—Give the eligibility criteria, and the sources and methods of case ascertainment and control selection. Give the rationale for the choice of cases and controls<br>*Cross-sectional study*—Give the eligibility criteria, and the sources and methods of selection of participants	7	

For peer review only - http://bmjopen.bmj.com/site/about/guidelines.xhtml
For peer review only

		(*b*) *Cohort study*—For matched studies, give matching criteria and number of exposed and unexposed<br>*Case-control study*—For matched studies, give matching criteria and the number of controls per case	N/A	
Variables	7	Clearly define all outcomes, exposures, predictors, potential confounders, and effect modifiers. Give diagnostic criteria, if applicable	7-8	Outcome variables & Predictive factors sections
Data sources/ measurement	8*	For each variable of interest, give sources of data and details of methods of assessment (measurement). Describe comparability of assessment methods if there is more than one group	7-8	Data sources, Outcome variables & Predictive factors sections
Bias	9	Describe any efforts to address potential sources of bias	8	"To explore bias in data availability, descriptive statistics for key patient characteristics (age, sex, socioeconomic deprivation, ethnicity and comorbidities) were summarised for three groups: all patients, those with complete case data, and those who had a previous discharge."
Study size	10	Explain how the study size was arrived at	7/8	"The Trust's electronic health records (EHR) were used to identify elective knee surgeries, using a combination of OPCS4 procedure and surgical site codes"<br>"Complete case analysis was used throughout. The complete case sample was n=2295, or 76.3% of the whole sample."

Continued on next page

For peer review only

Quantitative variables	11	Explain how quantitative variables were handled in the analyses. If applicable, describe which groupings were chosen and why	7-8	Outcome variables & Predictive factors sections
Statistical methods	12	(*a*) Describe all statistical methods, including those used to control for confounding	8-9	Statistical analysis section
		(*b*) Describe any methods used to examine subgroups and interactions	8-9	Statistical analysis section
		(*c*) Explain how missing data were addressed	8-9	Statistical analysis section
		(*d*) *Cohort study*—If applicable, explain how loss to follow-up was addressed<br>*Case-control study*—If applicable, explain how matching of cases and controls was addressed<br>*Cross-sectional study*—If applicable, describe analytical methods taking account of sampling strategy		
		(*e*) Describe any sensitivity analyses	8-9	Statistical analysis section
**Results**				
Participants	13*	(a) Report numbers of individuals at each stage of study—eg numbers potentially eligible, examined for eligibility, confirmed eligible, included in the study, completing follow-up, and analysed	10	"Between January 2016 and December 2019, 3008 primary elective knee replacements were performed. Of these, 2295 (76.3%) patients had data available for all variables and were included in the complete case analysis (see Figure 1). As the continuous length of stay outcome measure was capped at 30 days, the multivariable analyses for this outcome included 2274 patients"
		(b) Give reasons for non-participation at each stage		
		(c) Consider use of a flow diagram	Fig 1	
Descriptive data	14*	(a) Give characteristics of study participants (eg demographic, clinical, social) and information on exposures and potential confounders	Table 1	
		(b) Indicate number of participants with missing data for each variable of interest	Table 1	
		(c) *Cohort study*—Summarise follow-up time (eg, average and total amount)		
Outcome data	15*	*Cohort study*—Report numbers of outcome events or summary measures over time		
		Case-control study—Report numbers in each exposure category, or summary measures of exposure		

For peer review only

		Cross-sectional study—Report numbers of outcome events or summary measures	10	"The mean length of stay for patients who were in hospital for 30 days or less was 5.0 days (SD = 3.9), 15.4% of patients stayed in hospital for more than 7 days. Most patients (92.9%) did not remain in hospital once they were medically fit for discharge, but 7.1% did have a recorded medically fit for discharge date which preceded their discharge date."
Main results	16	(*a*) Give unadjusted estimates and, if applicable, confounder-adjusted estimates and their precision (eg, 95% confidence interval). Make clear which confounders were adjusted for and why they were included	Table 2	
		(*b*) Report category boundaries when continuous variables were categorized		
		(*c*) If relevant, consider translating estimates of relative risk into absolute risk for a meaningful time period		

Continued on next page

For peer review only

Other analyses	17	Report other analyses done—eg analyses of subgroups and interactions, and sensitivity analyses	Supplementary materials	
**Discussion**				
Key results	18	Summarise key results with reference to study objectives	11	Summary of main findings section
Limitations	19	Discuss limitations of the study, taking into account sources of potential bias or imprecision. Discuss both direction and magnitude of any potential bias	12	Strengths and limitations section
Interpretation	20	Give a cautious overall interpretation of results considering objectives, limitations, multiplicity of analyses, results from similar studies, and other relevant evidence	13	Implications for practice section
Generalisability	21	Discuss the generalisability (external validity) of the study results	12	Strengths and limitations section
**Other information**				
Funding	22	Give the source of funding and the role of the funders for the present study and, if applicable, for the original study on which the present article is based	3 & 9	Funding section & Role of funding sections

*Give information separately for cases and controls in case-control studies and, if applicable, for exposed and unexposed groups in cohort and cross-sectional studies.

Note: An Explanation and Elaboration article discusses each checklist item and gives methodological background and published examples of transparent reporting. The STROBE checklist is best used in conjunction with this article (freely available on the Web sites of PLoS Medicine at http://www.plosmedicine.org/, Annals of Internal Medicine at http://www.annals.org/, and Epidemiology at http://www.epidem.com/). Information on the STROBE Initiative is available at www.strobe-statement.org.

For peer review only

For peer review only - http://bmjopen.bmj.com/site/about/guidelines.xhtml

BMJ Open

Identification of risk factors associated with prolonged hospital stay following primary knee replacement surgery: a retrospective, longitudinal observational study

Journal:	*BMJ Open*
Manuscript ID	bmjopen-2022-068252.R1
Article Type:	Original research
Date Submitted by the Author:	16-Nov-2022
Complete List of Authors:	wilson, rebecca ; University of Bristol,<br>Margelyte, Ruta; University of Bristol, Translational Health Sciences, Bristol Medical School<br>Redaniel, Maria Theresa; University Hospitals Bristol and Weston NHS Foundation Trust, The National Institute for Health Research Applied Research Collaboration West (NIHR ARC West); University of Bristol, Population Health Sciences, Bristol Medical School<br>Eyles, Emily; University Hospitals Bristol and Weston NHS Foundation Trust, The National Institute for Health Research Applied Research Collaboration West (NIHR ARC West); University of Bristol, Population Health Sciences, Bristol Medical School<br>Jones, Tim; University Hospitals Bristol and Weston NHS Foundation Trust, The National Institute for Health Research Applied Research Collaboration West (NIHR ARC West); University of Bristol, Population Health Sciences, Bristol Medical School<br>Penfold, Chris; University Hospitals Bristol and Weston NHS Foundation Trust, The National Institute for Health Research Applied Research Collaboration West (NIHR ARC West); University of Bristol, Population Health Sciences, Bristol Medical School<br>Blom, AW; University of Bristol,<br>Elliott, Andrew; North Bristol NHS Trust<br>Harper, Alison; University of Exeter, The National Institute for Health Research Applied Research Collaboration South-West Peninsula (PenARC); University of Exeter, Medical School<br>Keen, Tim; North Bristol NHS Trust<br>Pitt, Martin; University of Exeter, The National Institute for Health Research Applied Research Collaboration South-West Peninsula (PenARC); University of Exeter, Medical School<br>Judge, Andrew; University of Bristol, Translational Health Sciences
**Primary Subject Heading**:	Surgery
Secondary Subject Heading:	Health services research
Keywords:	Knee < ORTHOPAEDIC & TRAUMA SURGERY, Adult orthopaedics < ORTHOPAEDIC & TRAUMA SURGERY, RHEUMATOLOGY, STATISTICS & RESEARCH METHODS, Orthopaedic & trauma surgery < SURGERY

SCHOLARONE™
Manuscripts

I, the Submitting Author has the right to grant and does grant on behalf of all authors of the Work (as defined in the below author licence), an exclusive licence and/or a non-exclusive licence for contributions from authors who are: i) UK Crown employees; ii) where BMJ has agreed a CC-BY licence shall apply, and/or iii) in accordance with the terms applicable for US Federal Government officers or employees acting as part of their official duties; on a worldwide, perpetual, irrevocable, royalty-free basis to BMJ Publishing Group Ltd ("BMJ") its licensees and where the relevant Journal is co-owned by BMJ to the co-owners of the Journal, to publish the Work in this journal and any other BMJ products and to exploit all rights, as set out in our licence.

The Submitting Author accepts and understands that any supply made under these terms is made by BMJ to the Submitting Author unless you are acting as an employee on behalf of your employer or a postgraduate student of an affiliated institution which is paying any applicable article publishing charge ("APC") for Open Access articles. Where the Submitting Author wishes to make the Work available on an Open Access basis (and intends to pay the relevant APC), the terms of reuse of such Open Access shall be governed by a Creative Commons licence – details of these licences and which Creative Commons licence will apply to this Work are set out in our licence referred to above.

Other than as permitted in any relevant BMJ Author's Self Archiving Policies, I confirm this Work has not been accepted for publication elsewhere, is not being considered for publication elsewhere and does not duplicate material already published. I confirm all authors consent to publication of this Work and authorise the granting of this licence.

For peer review only

Identification of risk factors associated with prolonged hospital stay following primary knee replacement surgery: a retrospective, longitudinal observational study

For peer review only

Rebecca Wilson, PhD[1,2]; Ruta Margelyte, MSc[3]; Maria Theresa Redaniel, PhD[1,2]; Emily Eyles, PhD[1,2]; Tim Jones, PhD[1,2]; Chris Penfold, PhD[1,2]; Ashley Blom, PhD[3]; Andrew Elliott, MSc[4]; Alison Harper, PhD[5,6]; Tim Keen, MSc[4]; Martin Pitt, EngD[5,6]; Andrew Judge, PhD[3]

1. The National Institute for Health Research Applied Research Collaboration West (NIHR ARC West) at University Hospitals Bristol and Weston NHS Foundation Trust, Bristol, UK
2. Population Health Sciences, Bristol Medical School, University of Bristol, Bristol, UK
3. Translational Health Sciences, Bristol Medical School, University of Bristol, Bristol, UK
4. North Bristol NHS Trust, Bristol, UK
5. The National Institute for Health Research Applied Research Collaboration South-West Peninsula (PenARC), University of Exeter Medical School, Exeter, UK
6. Medical School, University of Exeter, Exeter, UK

Correspondence to:

Dr Rebecca Wilson

National Institute for Health Research Applied Research Collaboration West (NIHR ARC West)

University Hospitals Bristol NHS Foundation Trust

9th Floor, Whitefriars, Lewins Mead, Bristol, BS1 2NT

For peer review only - http://bmjopen.bmj.com/site/about/guidelines.xhtml

rebecca.wilson@bristol.ac.uk

For peer review only

For peer review only - http://bmjopen.bmj.com/site/about/guidelines.xhtml

Abstract

Objectives

To identify risk factors associated with prolonged length of hospital stay and staying in hospital longer than medically necessary following primary knee replacement surgery.

Design

Retrospective, longitudinal observational study.

Setting

Elective knee replacement surgeries between 2016-2019 were identified using routinely collected data from an NHS Trust in England.

Participants

There were 2295 knee replacement patients with complete data included in analysis. The mean age was 68 (standard deviation 11) and 60% were female.

Outcome measures

We assessed a binary length of stay outcome (>7 days), a continuous length of stay outcome (≤30 days) and a binary measure of whether patients remained in hospital when they were medically fit for discharge.

Results

The mean length of stay was 5.0 days (SD 3.9), 15.4% of patients were in hospital for >7 days and 7.1% remained in hospital when they were medically fit for discharge. Longer length of stay was associated with older age (*b*=0.08, 95% CI 0.07 to 0.09), female sex (*b*=0.36, 95% CI 0.06 to 0.67), high deprivation (*b*=0.98, 95% CI 0.47 to 1.48) and more comorbidities (*b*=2.48, 95% CI 0.15 to 4.81). Remaining in hospital beyond being medically fit for discharge was associated with older age

For peer review only

(OR=1.07, 95% CI 1.05 to 1.09), female sex (OR=1.71, 95% CI 1.19 to 2.47) and high deprivation (OR=2.27, 95% CI 1.27 to 4.06).

Conclusions

The regression models could be used to identify which patients are likely to occupy hospital beds for longer. This could be helpful in scheduling operations to aid hospital efficiency by planning these patients' operations for when the hospital is less busy.

For peer review only

Strengths and limitations of this study

- Robust statistical methods used to develop statistical models with good discrimination and calibration.
- Consistent findings across three output measures of hospital throughput.
- Single centre study may restrict generalisability of findings.
- Data do not include operations during or after the COVID-19 pandemic, after which the healthcare landscape changed and waiting list times have increased further.

Word count: 2367

For peer review only - http://bmjopen.bmj.com/site/about/guidelines.xhtml

Introduction

Osteoarthritis is a common disease and a leading cause of disability worldwide (1), with pain being the primary reason that people seek medical care. In those with end stage disease, joint replacement surgery is a well-established, common and highly effective surgical procedure (2), where the majority of patients achieve substantial reductions in pain, improved joint function, mobility and health related quality of life (3). These operations cost the National Health Service (NHS) approximately £2 billion annually, which represents almost 1.5% of the entire NHS budget (4). Total knee replacements are a common surgery in the UK, with up to 100,000 performed each year (5) and with a lifetime risk of 10.8% for women and 8.1% for men undergoing knee replacement (6). The number of knee replacement surgeries are increasing in number due to an ageing and increasingly obese population (7,8) and are expected to continue to rise by 40% over the next 40 years (9,10).

Waiting times for NHS care have increased in recent years (11), whilst hospitals' capacity (the number of available theatres, surgeons and beds) is declining (7). NHS acute hospital trusts face challenges in planning hospital bed capacity, especially in winter months when patient demand for hospital care is very high. In the UK, prior to the COVID-19 pandemic, substantial challenges existed for delivering timely joint replacement surgery, with orthopaedics having the largest waiting list of any individual surgical specialty (12). Furthermore, planned operations are commonly cancelled, with 60% of cancellations happening on the day of surgery, usually due to lack of beds, staff, and/or operating capacity, which can be compounded during the winter months (13).

Patients remaining in hospital for longer than is necessary following surgery results in fewer hospital beds being available for the next scheduled patients. The time patients should expect to remain in hospital following total knee replacement surgery has decreased in recent years (from 3.7 days to 3.0 days between 2006 and 2016) (14), which suggests that hospital capacity and waiting lists could

For peer review only - http://bmjopen.bmj.com/site/about/guidelines.xhtml

have scope to improve. Furthermore, shorter length of hospital stay does not reflect compromised patient care (15) and is financially more sustainable.

Waiting lists could be reduced if hospitals are able to plan the timing of surgeries in order to optimise hospital efficiency. There is a need to identify predictors of hospital throughput, to better plan elective surgery, by understanding the characteristics of patients at greatest risk of staying in hospital longer than medically necessary.

The aim of this study was to identify risk factors associated with prolonged length of hospital stay and staying in hospital longer than medically necessary following primary knee replacement surgery using linked routinely collected data from an NHS Trust in South-West England.

For peer review only

Methods

Data sources

This is a longitudinal observational study and is reported in line with the STROBE reporting guidelines (16). Routinely collected data from an NHS Trust in England were used to identify patients receiving elective primary total knee replacement surgeries between 2016 and 2019. The Trust's electronic health records (EHR) were used to identify elective total knee replacements, using a combination of OPCS4 procedure and surgical site codes (see Supplementary Material Table S1). Tourniquet and wound drainage were not routinely used. Tranexamic acid was routinely administered intravenously intraoperatively. Further, the EHR were used to extract data for patient demographics (age, sex, deprivation quintile and comorbidities), and for admission details (length of stay and time and date of admission and discharge). Information describing the Trust's ratio of emergency to elective surgeries, daily emergency (non-elective) admissions and daily non-elective occupied beds were obtained from the Hospital Episode Statistics admitted patient care (HES-APC) dataset (17).

Outcome variables

A continuous measure of length of stay was generated to summarise the number of days between admission and discharge, up to 30 days; those in hospital for more than 30 days were coded as missing for this variable (this was to exclude outliers and excluded only 0.92% (N=21) of complete cases from analysis). Two binary outcome variables were derived, one indicating if patient's hospital stay was longer than 7 days (0= ≤7 days, 1= >7 days) and one indicating if patient had a recorded medically fit for discharge date that preceded their discharge date (0= did not have a medically fit for discharge date recorded, 1= patient remained in hospital when they were medically fit for discharge). Patients without a medically fit for discharge date were assumed to have been discharged when medically fit.

For peer review only - http://bmjopen.bmj.com/site/about/guidelines.xhtml

Predictive factors

Variables in the multivariable models include: a continuous measure of age on admission, patient sex (0= male, 1= female), patient area level deprivation (IMD quintile derived from LSOA (18)), comorbidities (as measured by the weighted Charlson Comorbidity Index (19)), a categorical measure of time since last discharge (0-2 months, 2-12 months, 12 months or more, or no previous admissions), a categorical measure of admission hour (06.00-12.00, 12.00-18.00, 18.00-06.00), the day of the week, season (winter: December-February; spring: March-May; summer: June-August; autumn: September-November) and year of admission, and the Trust monthly emergency/elective admissions ratio, daily non-elective admissions, and daily non-elective occupied beds.

Statistical analysis

Sample characteristics were summarised for all patients. To explore bias in data availability, descriptive statistics for key patient characteristics (age, sex, socioeconomic deprivation, ethnicity and comorbidities) were summarised for three groups: all patients, those with complete case data, and those who had a previous discharge. No notable differences in these characteristics were observed between these subsamples (see Table S2, Supplementary material). Complete case analysis was used throughout. The complete case sample was n=2295, or 76.3% of the whole sample.

Univariable and multivariable regression models were used to test associations between predictive factors and outcomes. Model assumptions were checked, and the following interactions were tested for: age and sex, age and comorbidities, age and season, age and deprivation, season and comorbidities, deprivation and comorbidities, season and deprivation. No interactions were observed. There was some evidence to suggest the continuous outcome (length of stay) violated the assumptions for the linear regression model (residuals not normally distributed), so robust standard errors were estimated using the Huber-White sandwich estimator (20).

For peer review only - http://bmjopen.bmj.com/site/about/guidelines.xhtml

Multivariable logistic regression models were used for binary outcomes and linear regression for the continuous outcome. The same 12 potential predictors (age, sex, area-level deprivation, comorbidities, days since last discharge, elective/non-elective ratio, non-elective admissions, non-elective occupied beds, admission hour and year, day and season of admission) were entered into the models for all outcomes. For each outcome, we ran least absolute shrinkage and selection operator (LASSO), elastic-net and ridge regression models. To produce the final models, we performed multivariable logistic (for binary outcomes)/linear (for continuous outcome) regression using backwards variable selection at p=0.1 and obtained C-statistics for the binary outcomes and R^2 for the continuous outcome. Calibration plots were produced for the final models for the binary outcomes.

Using the predictor variables retained in the backwards selection regression models, forest plots were produced to display the effects of each predictive factor for each outcome. All analyses were performed using Stata 17 (21).

Patient and public involvement

Patients and the public were consulted in a workshop for suggestions and comments to inform the development of the grant. They were again consulted in a follow-up workshop where initial results were presented. They were asked about their opinion on the prioritisation of particular patient groups (e.g. less complex patients during winter and complex patients in the summer) to maximise efficiency for surgery waiting lists.

Ethics approval

We were provided with pseudonymised North Bristol Trust hospital admissions data under the NIHR ARC West Partnership Agreement. The project received ethical approval from the University of Bristol Faculty of Health Sciences ethical review board on 3rd November 2020 (ref# 109024).

For peer review only - http://bmjopen.bmj.com/site/about/guidelines.xhtml

For peer review only

We were provided with routinely collected Hospital Episode Statistics data under licence from NHS Digital (DARS-NIC-17875-X7K1V). The licence allows us to use the information under Section 261 of the Health and Social Care Act 2012, 2(b)(ii): "after taking into account the public interest as well as the interests of the relevant person, considers that it is appropriate for the information to be disseminated".

Role of the funding source

The funders had no role in any of the following: the study design; the collection, analysis, and interpretation of data; the writing of the report; the decision to submit the paper for publication.

For peer review only

For peer review only - http://bmjopen.bmj.com/site/about/guidelines.xhtml

Results

Between January 2016 and December 2019, 3008 primary elective knee replacements were performed. Of these, 2295 (76.3%) patients had data available for all variables and were included in the complete case analysis (see Figure 1). As the continuous length of stay outcome measure was capped at 30 days, the multivariable analyses for this outcome included 2274 patients. Descriptive statistics are presented in Table 1 for the complete case sample. The mean length of stay for patients who were in hospital for 30 days or less was 5.0 days (SD = 3.9), 15.4% of patients stayed in hospital for more than 7 days. Most patients (92.9%) did not remain in hospital once they were medically fit for discharge, but 7.1% did have a recorded medically fit for discharge date which preceded their discharge date.

Multivariable backwards regression models are presented in Table 2 and in Figures 2-4. The full multivariable regression models, LASSO, elastic net and ridge regression models for each outcome are presented in Supplementary Tables S3-S5. The models for the binary outcomes (length of stay and medically fit for discharge) are accompanied by calibration plots (Figures S1-S4).

Remaining in hospital for more than seven days following surgery was associated with older age, higher deprivation, more comorbidities and being recently discharged from hospital (see Table 2/Figure 2). Associations between length of stay and time of admission were also observed but the confidence intervals were wide. Year and day of admission were also associated with length of the stay but without any apparent trend. No associations were found for sex, season of admission or hospital related factors (emergency over elective admissions ratio, non-elective admissions ratio or non-elective occupied beds).

Similarly, longer length of stay following surgery (measured using a continuous variable ranging from 0 to 30 days) was associated with older age, increasing deprivation, more comorbidities and recently being discharged from hospital (compared with never having been admitted to hospital or being

For peer review only

discharged from hospital at least over two months ago). Female sex was also associated with the continuous measure of length of stay (see Table 2/Figure 3). A spurious association with a wide confidence interval was again observed for admission hour. No associations were observed for hospital related factors, admission year, day or season.

Remaining in hospital when being medically fit for discharge was associated with older age, being female, greater deprivation, more comorbidities (though not the highest category of comorbidities, which was dropped from the model), being discharged from hospital recently (compared with 12 months or more or never), surgeries in 2017 and 2018 (compared with 2016). A weak association was observed with admission hour and no associations were found for hospital related factors, admission day or season (Table 2/Figure 4).

The calibration plots (Figures 5-6) indicate close agreement for predicted and observed values for the binary length of stay and the medically fit for discharge outcome. The respective C-statistics of 0.72 and 0.74 also indicated good model discrimination.

Discussion

Summary of main findings

In this study, we observed that over 7% of patients remained in hospital beyond the date they were medically fit to be discharged, and 15% had long stays in hospital of more than 7 days. Being able to reduce this burden on hospital throughput would have a positive impact on overall hospital efficiency. Risk factors associated with remaining in hospital longer, and for longer than is necessary, included: older age, female patients with more comorbidities and those recently admitted to hospital. Patients from more deprived areas also had worse outcomes. There was no evidence of interactions with season, suggesting that efforts to limit operations to older patients and those with co-morbidities to summer months will not impact on hospital efficiency and throughput.

For peer review only - http://bmjopen.bmj.com/site/about/guidelines.xhtml

For peer review only

What is already known

Our results were in line with previous research showing longer length of stay is associated with older age, being female and worse health (14,22–24). Hospital and surgeon related factors (including hospital site, surgeon, time and day of surgery) (22) were also cited as predictors of length of stay, some of which are modifiable and presents opportunities to optimise efficiency, however, we found that hospital-related factors were not predictors of length of stay, although variables in this study, such as admission hour and day, were limited by small numbers in some categories. The factors associated with outcomes in our study were all patient related, including health-related and sociodemographic.

Strengths and limitations

This observational study had many strengths, including its reasonable sample size and use of robust statistical methods. This allowed us to identify a number of predictors with good discrimination and calibration. We also utilised three metrics of throughput as outcomes which demonstrated consistent findings. There are of course limitations to our study. There is great variation in practice and provision of services across the country (7) and, as a single centre study, the results may not be generalisable to other NHS trusts and settings. We only included data on operations performed in the NHS Trust and not privately funded patients. We purposefully analysed data from before the start of the COVID-19 pandemic, after which the healthcare landscape changed extraordinarily. Waiting times for knee replacement surgery have since increased, leading to huge burden on patients' wellbeing (25), and this is not represented in our results. There were missing data, however

For peer review only - http://bmjopen.bmj.com/site/about/guidelines.xhtml

comparison of patient characteristics of those in full versus complete case dataset shows no evidence of selection (responder) bias (see Supplementary Table S2).

Implications for practice

Our models would allow health care providers to identify which patients are more likely to remain in hospital for longer, using these known variables, or risk factors. Whilst providers would not be able to change these risk factors, this knowledge could be used in planning surgeries and hospital capacity, potentially allowing the more complex surgeries with a longer predicted in-patient stay to be scheduled for when the hospital is under less strain, e.g., in the summer months.

Rescheduling patients' surgeries due to personal characteristics could be considered controversial, or potentially introducing inequalities. However, when asked if they would mind if surgeries were scheduled to a particular time based on patient characteristics, our PPI group said not, providing they were kept informed and could rely on the information being told to them. If there is evidence to support rescheduling patients in order to improve efficiency, patients and their caregivers could accept surgeries being moved.

We were able to build good-fitting models of length of stay and staying in hospital beyond being medically fit for discharge using data from all total knee replacements between 2016 and 2019 which can identify which patients are more likely to stay in hospital for longer and for longer than is necessary. These models may be used in planning bed capacity and potentially reduce waiting list times.

For peer review only

Contributors

RW, RM, MTR, and AJ drafted the original manuscript. RW and RM analysed the data. RW, RM, MTR, EE, TJ CP, AB, AE, AH, TK, MP and AJ all contributed to the conception and planning of this project and all reviewed and revised the manuscript. MTR and AJ supervised the project.

Competing interest

We declare no competing interests.

Funding

This study was funded by the Health Data Research (HDR) UK South West Better Care Partnership (#6.12). This research was supported by the National Institute for Health Research (NIHR) Applied Research Collaboration West (ARC West) at University Hospitals Bristol and Weston NHS Foundation Trust (core NIHR infrastructure funded: NIHR200181).

Data availability statement

Data may be obtained from a third party and are not publicly available. The data used in the study are collected by the North Bristol Trust (NBT) as part of their care and support. Sharing of anonymised data with the University of Bristol was underpinned by a data sharing agreement and solely covers the purposes of this study. Data requests can be made through the NBT.

For peer review only - http://bmjopen.bmj.com/site/about/guidelines.xhtml

References

1. Vos T, Flaxman AD, Naghavi M, Lozano R, Michaud C, Ezzati M, et al. Years lived with disability (YLDs) for 1160 sequelae of 289 diseases and injuries 1990–2010: a systematic analysis for the Global Burden of Disease Study 2010. Lancet. 2012 Dec 15;380(9859):2163–96.

2. National Institute for Health and Care Excellence. Osteoarthritis: care and management (CG177) [Internet]. Nice Guideline. 2014 [cited 2022 Jun 22]. Available from: https://www.nice.org.uk/guidance/cg177/chapter/Recommendations#referral-for-consideration-of-joint-surgery-2

3. Ethgen O, Bruyerè O, Richy F, Dardennes C, Reginster JY. Health-Related Quality of Life in Total Hip and Total Knee Arthroplasty: A Qualitative and Systematic Review of the Literature. J Bone Jt Surg. 2004;86(5):963–74.

4. National Institute for Health and Care Excellence. Joint replacement (primary): hip, knee and shoulder [Internet]. 2020. Available from: https://www.nice.org.uk/guidance/ng157/resources/joint-replacement-primary-hip-knee-and-shoulder-pdf-66141845322181

5. Price AJ, Alvand A, Troelsen A, Katz JN, Hooper G, Gray A, et al. Knee replacement. Vol. 392, The Lancet. Lancet Publishing Group; 2018. p. 1672–82.

6. Culliford DJ, Maskell J, Kiran A, Judge A, Javaid MK, Cooper C, et al. The lifetime risk of total hip and knee arthroplasty: results from the UK general practice research database. Osteoarthr Cartil. 2012 Jun 1;20(6):519–24.

7. Briggs T. A national review of adult elective orthopaedic services in England: Getting it right first time [Internet]. 2015 [cited 2022 May 17]. Available from: https://gettingitrightfirsttime.co.uk/wp-content/uploads/2017/06/GIRFT-National-Report-Mar15-Web.pdf

8. Culliford DJ, Maskell J, Beard DJ, Murray DW, Price AJ, Arden NK. Temporal trends in hip and knee replacement in the United Kingdom: 1991 to 2006. J Bone Jt Surg - Ser B [Internet]. 2010 Jan 1 [cited 2022 May 17];92(1):130–5. Available from: https://online.boneandjoint.org.uk/doi/abs/10.1302/0301-620X.92B1.22654

9. Culliford D, Maskell J, Judge A, Cooper C, Prieto-Alhambra D, Arden NK. Future projections of total hip and knee arthroplasty in the UK: Results from the UK Clinical Practice Research Datalink. Osteoarthr Cartil. 2015 Apr 1;23(4):594–600.

10. Matharu G, Culliford D, Blom A, Judge A. Projections for primary hip and knee replacement surgery up to the year 2060: an analysis based on data from The National Joint Registry for England, Wales, Northern Ireland and the Isle of Man. Ann R Coll Surg Engl [Internet]. 2022 Jun [cited 2022 Jun 22];104(6):443–8. Available from: https://pubmed.ncbi.nlm.nih.gov/34939832/

11. Anandaciva S. Hidden from the targets : how long are patients waiting for NHS care ? [Internet]. The King's Fund. 2018 [cited 2022 May 17]. Available from: https://www.kingsfund.org.uk/blog/2018/02/hidden-targets-patients-waiting-nhs-care

12. British Orthopaedic Association. A message for people waiting for joint replacement and other orthopaedic surgery [Internet]. 2020 [cited 2022 Jun 22]. Available from: https://www.boa.ac.uk/resources/a-message-for-people-waiting-for-joint-replacement-and-

For peer review only - http://bmjopen.bmj.com/site/about/guidelines.xhtml

other-orthopaedic-surgery.html

13. Royal College of Surgeons. Lack of NHS beds halted vital operations last year [Internet]. 2020 [cited 2022 Jun 22]. Available from: https://www.rcseng.ac.uk/news-and-events/media-centre/press-releases/rcs-survey-lack-of-nhs-beds-halted-operations/

14. Sarpong NO, Boddapati V, Herndon CL, Shah RP, Cooper HJ, Geller JA. Trends in Length of Stay and 30-Day Complications After Total Knee Arthroplasty: An Analysis From 2006 to 2016. J Arthroplasty [Internet]. 2019 Aug 1 [cited 2022 May 16];34(8):1575–80. Available from: https://www.sciencedirect.com/science/article/pii/S0883540319303596?casa_token=b9fcXF6p0ccAAAAA:L_PRg0vfPDLJGruYME1HOUHgZJTwj893jR_Osd-FaddD3ONJRdXitJOrUKfSU7SpzaG14Q4

15. Sutton JC, Antoniou J, Epure LM, Huk OL, Zukor DJ, Bergeron SG. Hospital discharge within 2 days following total hip or knee arthroplasty does not increase major-complication and readmission rates. J Bone Jt Surg - Am Vol [Internet]. 2016 [cited 2022 May 17];98(17):1419–28. Available from: https://journals.lww.com/jbjsjournal/Fulltext/2016/09070/Hospital_Discharge_within_2_Days_Following_Total.1.aspx

16. Vandenbroucke J, Von Elm E, Altman D, Gøtzsche P, Mulrow C, Pocock S. Strengthening the Reporting of Observational Studies in Epidemiology (STROBE): explanation and elaboration. Ann Intern Med. 2007;147(8):573–8.

17. Herbert A, Wijlaars L, Zylbersztejn A, Cromwell D, Hardelid P. Data Resource Profile: Hospital Episode Statistics Admitted Patient Care (HES APC). Int J Epidemiol [Internet]. 2017 Aug 1 [cited 2022 Aug 8];46(4):1093. Available from: /pmc/articles/PMC5837677/

18. Payne R, Abel G. UK indices of multiple deprivation - a way to make comparisons across constituent countries. London: Health Statistics Quarterly Office for National Statistics; 2012.

19. Charlson M, Szatrowski TP, Peterson J, Gold J. Validation of a combined comorbidity index. J Clin Epidemiol. 1994 Nov 1;47(11):1245–51.

20. Kirkwood BR, Sterne JA. Essential Medical Statistics. 2nd Editio. Blackwell Publishing Ltd; 2010.

21. StataCorp. Stata Statistical Software: Release 17. College Station, TX: StataCorp LLC; 2021.

22. Piuzzi NS, Strnad GJ, Ali Sakr Esa W, Barsoum WK, Bloomfield MR, Brooks PJ, et al. The Main Predictors of Length of Stay after Total Knee Arthroplasty: Patient-Related or Procedure-Related Risk Factors. J Bone Jt Surg - Am Vol [Internet]. 2019 Jun 19 [cited 2022 May 17];101(12):1093–101. Available from: https://journals.lww.com/jbjsjournal/Fulltext/2019/06190/The_Main_Predictors_of_Length_of_Stay_After_Total.7.aspx

23. Husted H, Holm G, Jacobsen S. Predictors of length of stay and patient satisfaction after hip and knee replacement surgery: Fast-track experience in 712 patients. Acta Orthop [Internet]. 2008;79(2):168–73. Available from: https://www.tandfonline.com/action/journalInformation?journalCode=iort20

24. Sephton BM, Bakhshayesh P, Edwards TC, Ali A, Kumar Singh V, Nathwani D. Predictors of extended length of stay after unicompartmental knee arthroplasty. J Clin Orthop Trauma. 2020 Mar 1;11:S239–45.

25. Clement ND, Scott CEH, Murray JRD, Howie CR, Deehan DJ, Farrow L, et al. The number of patients "worse than death" while waiting for a hip or knee arthroplasty has nearly doubled

For peer review only - http://bmjopen.bmj.com/site/about/guidelines.xhtml

during the COVID-19 pandemic. https://doi.org/101302/0301-620X103BBJJ-2021-0104R1 [Internet]. 2021 Mar 23 [cited 2022 May 17];103-B(4):672–80. Available from: https://online.boneandjoint.org.uk/doi/abs/10.1302/0301-620X.103B.BJJ-2021-0104.R1

For peer review only

For peer review only - http://bmjopen.bmj.com/site/about/guidelines.xhtml

Tables

Table 1: Characteristics of complete cases who have undergone NBT elective admissions for primary knee replacements (2015-2019) (N=2295)

Variable	N (%)[1]	Proportion (%) admitted >7 days	Mean length of stay (SD) N=2274	Proportion (%) medically fit for discharge
**Age at admission**				
Age at admission, mean (SD)	67.4 (11.3)	-	-	-
**Sex**				
Female	1381 (60.2)	15.3	5.1 (3.9) (N=1367)	8.5
Male	914 (39.8)	15.5	4.7 (3.8) (N=907)	5.0
**Deprivation (IMD score)**				
1 (least deprived)	588 (25.6)	11.1	4.4 (3.1) (N=583)	
2	564 (24.6)	18.3	5.1 (4.0) (N=555)	
3	395 (17.2)	17.0	5.2 (4.5) (N=391)	
4	418 (18.2)	14.8	5.0 (3.7) (N=416)	
5 (most deprived)	330 (14.4)	17.0	5.2 (4.2) (N=329)	
**Ethnicity[2]**				
Non-White	76 (5.3)	-	-	-
Asian	25 (1.7)	-	-	-
Black	31 (2.2)	-	-	-
Mixed	10 (0.7)	-	-	-
Other	10 (0.7)	-	-	-
White	1368 (94.7)	-	-	-
Unknown	851	-	-	-
**Comorbidities (weighted Charlson index)**				
0	1260 (54.9)	12.0	4.5 (3.4) (N=1255)	4.6
1-2	867 (37.8)	17.9	5.4 (4.1) (N=854)	9.5
3-4	148 (6.5)	26.4	6.6 (5.3) (N=145)	15.5
>=5	20 (0.9)	8.0	7.8 (5.5)	0
**Time since last discharge**				
0-2 months	338 (14.7)	26.9	6.3 (5.0) (N=332)	
2-12 months	474 (20.7)	18.8	5.5 (4.2) (N=470)	
12 months or more	409 (17.8)	13.0	4.6 (3.4)	
never	1074 (46.8)	11.1	4.4 (3.3) (N=1063)	
**Emergency over elective admissions ratio (general/acute, monthly)**, Mean ± SD	0.8 ± 0.1	-	-	-
**Emergency admissions, daily**, Mean ± SD	152.6 ± 21.2	-	-	-
**Emergency occupied beds, daily**, Mean ± SD	914.9 ± 43.4	-	-	-
**Admission hour category**				
24.00-06.00	2 (0.1)	50.0	9.5 (3.5)	0
06.00-12.00	2092 (91.2)	14.4	4.9 (3.7) (N=2075)	6.7
12.00-18.00	195 (8.5)	22.6	5.6 (4.7) (N=192)	9.7
18.00-24.00	6 (0.3)	100.0	14.2 (7.0) (N=5)	50.0
**Year of Admission**				

For peer review only - http://bmjopen.bmj.com/site/about/guidelines.xhtml

2016	185 (8.1)	10.8	4.7 (3.1)	2.7
2017	783 (34.1)	15.6	4.9 (3.6) (N=770)	7.2
2018	629 (27.4)	18.4	5.3 (4.1) (N=626)	9.1
2019	698 (30.4)	13.6	4.8 (4.1) (N=693)	6.5
**Month of Admission**				
January	131 (5.7)	20.6	5.4 (4.7)	8.4
February	170 (7.4)	14.1	4.9 (4.3)	3.5
March	208 (9.1)	18.8	5.1 (4.2) (N=204)	7.2
April	183 (8.0)	17.5	4.9 (3.0) (N=181)	6.6
May	194 (8.5)	17.0	5.1 (3.8) (N=192)	9.3
June	194 (8.5)	9.8	4.4 (3.0) (N=192)	7.2
July	188 (8.2)	16.0	4.9 (4.1) (N=185)	10.6
August	164 (7.2)	15.2	4.9 (3.9) (N=161)	5.5
September	187 (8.2)	14.4	5.2 (4.1)	8.0
October	254 (11.1)	15.8	5.0 (4.0) (N=251)	5.9
November	260 (11.3)	13.5	4.9 (3.8) (N=258)	8.1
December	162 (7.1)	13.6	5.0 (3.5)	4.3
**Day of the Week of Admission**				
Sunday	2 (0.1)	50.0	8.5 (10.6)	50.0
Monday	495 (21.6)	18.4	4.8 (3.8) (N=490)	5.5
Tuesday	467 (20.4)	17.3	4.9 (3.7) (N=461)	8.8
Wednesday	403 (17.6)	13.2	5.0 (4.4) (N=399)	7.7
Thursday	408 (17.8)	14.2	5.0 (3.5) (N=406)	6.1
Friday	467 (20.4)	13.9	5.1 (3.9) (N=463)	7.5
Saturday	53 (2.3)	7.6	4.7 (3.2) (N=53)	5.7
**Season of Admission**				
Winter (Dec-Feb)	463 (20.2)	15.8	5.1 (4.1)	5.2
Spring (Mar-May)	585 (25.5)	17.8	5.0 (3.7) (N=577)	7.7
Summer (Jun-Aug)	546 (23.8)	13.6	4.7 (3.7) (N=538)	7.9
Autumn (Sep-Nov)	701 (30.5)	14.6	5.0 (3.9) (N=696)	7.3
**Spell length of stay**, Mean ± SD	5.5 ± 6.1	-	-	-
**Spell length of stay up to 30 days**, Mean ± SD, **(N=2274)**	5.0 ± 3.9	-	-	-
**Patients staying >7 days**				
<=7 (no)	1942 (84.6)	-	-	-
>7 (yes)	353 (15.4)	-	-	-
**Patients medically fit for discharge (MFFD)**				
with MFFD date before discharge	163 (7.1)	-	-	-
no MFFD date	2132 (92.9)	-	-	-
**Days between MFFD date and Discharge**, Mean ± SD, **(N=163)**	7.2 ± 13.7	-	-	-

[1] n (%) for categorical, mean ± SD for continuous variables

[2] Percentages are for known data. Not included in analysis due to large number of 'unknowns'

For peer review only

For peer review only - http://bmjopen.bmj.com/site/about/guidelines.xhtml

Table 2: Predictors of length of stay and remaining in hospital beyond being medically fit for discharge

Variable	Length of stay (admissions > 7 days) N=2295	Length of stay (≤ 30 days) N=2274[1]	Medically fit for discharge N=2275[2]
	OR (95% CI)	Coef (95% CI)	OR (95% CI)
**Age at admission**	1.06 (1.04 to 1.07)*	0.08 (0.07 to 0.09)*	1.07 (1.05 to 1.09)*
**Sex (female vs male)**	-	0.36 (0.06 to 0.67)*	1.71 (1.19 to 2.47)*
**Deprivation (IMD score)**			
1 (least deprived)	1.00	0.00	1.00
2	1.74 (1.22 to 2.47)*	0.65 (0.26 to 1.04)*	1.76 (1.05 to 2.96)*
3	1.74 (1.18 to 2.58)*	0.80 (0.32 to 1.28)*	1.84 (1.05 to 3.23)*
4	1.54 (1.04 to 2.28)*	0.75 (0.33 to 1.16)*	2.07 (1.20 to 3.57)*
5 (most deprived)	2.03 (1.34 to 3.05)*	0.98 (0.47 to 1.48)*	2.27 (1.27 to 4.06)*
**Comorbidities (weighted Charlson Comorbidities index)**			
0	1.00	0.00	1.00
1-2	1.40 (1.08 to 1.81)*	0.63 (0.31 to 0.94)*	1.89 (1.32 to 2.71)*
3-4	1.85 (1.20 to 2.86)*	1.42 (0.55 to 2.29)*	2.56 (1.48 to 4.42)*
≥5	3.03 (1.14 to 8.05)*	2.48 (0.15 to 4.81)*	-
**Time since Last Discharge**			
0-2 months	1.00	0.00	1.00
2-12 months	0.67 (0.47 to 0.95)*	-0.65 (-1.29 to -0.02)*	0.97 (0.60 to 1.57)
12 months or more	0.41 (0.27 to 0.61)*	-1.45 (-2.06 to -0.84)*	0.53 (0.30 to 0.92)*
Never	0.38 (0.28 to 0.53)*	-1.49 (-2.05 to -0.93)*	0.53 (0.33 to 0.84)*
**Emergency over elective admissions ratio**	-	-	-
**NEL admissions**	-	-	-

For peer review only - http://bmjopen.bmj.com/site/about/guidelines.xhtml

**NEL occupied beds**	-	-	-
**Admission hour category**			
06.00-12.00	1.00	0.00	1.00
12.00-18.00	1.81 (1.23 to 2.66)*	0.79 (0.17 to 1.41)*	1.56 (0.91 to 2.66)
18.00-06:00	77.21 (8.01 to 744.20)*	7.55 (2.85 to 12.24)*	8.11 (1.74 to 37.75)*
**Year of Admission**			
2016	1.00	0.00	1.00
2017	1.66 (0.96 to 2.86)	0.20 (-0.27 to 0.67)	2.95 (1.14 to 7.64)*
2018	1.96 (1.12 to 3.42)*	0.47 (-0.04 to 0.99)	3.89 (1.49 to 10.12)*
2019	1.14 (0.65 to 1.99)	-0.21 (-0.71 to 0.30)	2.32 (0.88 to 6.09)
**Day of Admission**			
Sunday	-	-	-
Monday	1.00	-	-
Tuesday	0.89 (0.62 to 1.26)	-	-
Wednesday	0.59 (0.40 to 0.88)*	-	-
Thursday	0.66 (0.45 to 0.97)*	-	-
Friday	0.78 (0.54 to 1.13)	-	-
Saturday	0.20 (0.06 to 0.73)*	-	-
**Season of Admission**			
Winter (Dec-Feb)	1.00	-	-
Spring (Mar-May)	1.14 (0.80 to 1.61)	-	-
Summer (Jun-Aug)	0.73 (0.50 to 1.06)	-	-
Autumn (Sep-Nov)	0.90 (0.64 to 1.28)	-	-
	**C-statistic (95% CI)**	**R^2**	**C-statistic (95% CI)**
	0.72 (0.69 to 0.74)	0.130	0.74 (0.71 to 0.78)

[1] included sample N=2274 patients with length of stay ≤30 days

[2] included sample N=2295 minus those excluded when CCI score ≥5 was dropped from the model (N=20)

For peer review only - http://bmjopen.bmj.com/site/about/guidelines.xhtml

Figure titles

Figure 1: Flow diagram of patients excluded and included in the study

Figure 2: Predictors of length of stay of more than seven days for patients following knee replacement surgery

Figure 3: Predictors of length of stay (using a continuous measure) in patients following knee replacement surgery

Figure 4: Predictors of remaining in hospital beyond being declared medically fit following knee replacement surgery

Figure 5: Calibration plot for final model for binary length of stay outcome

Figure 6: Calibration plot for final model for medically fit for discharge outcome

For peer review only

For peer review only - http://bmjopen.bmj.com/site/about/guidelines.xhtml

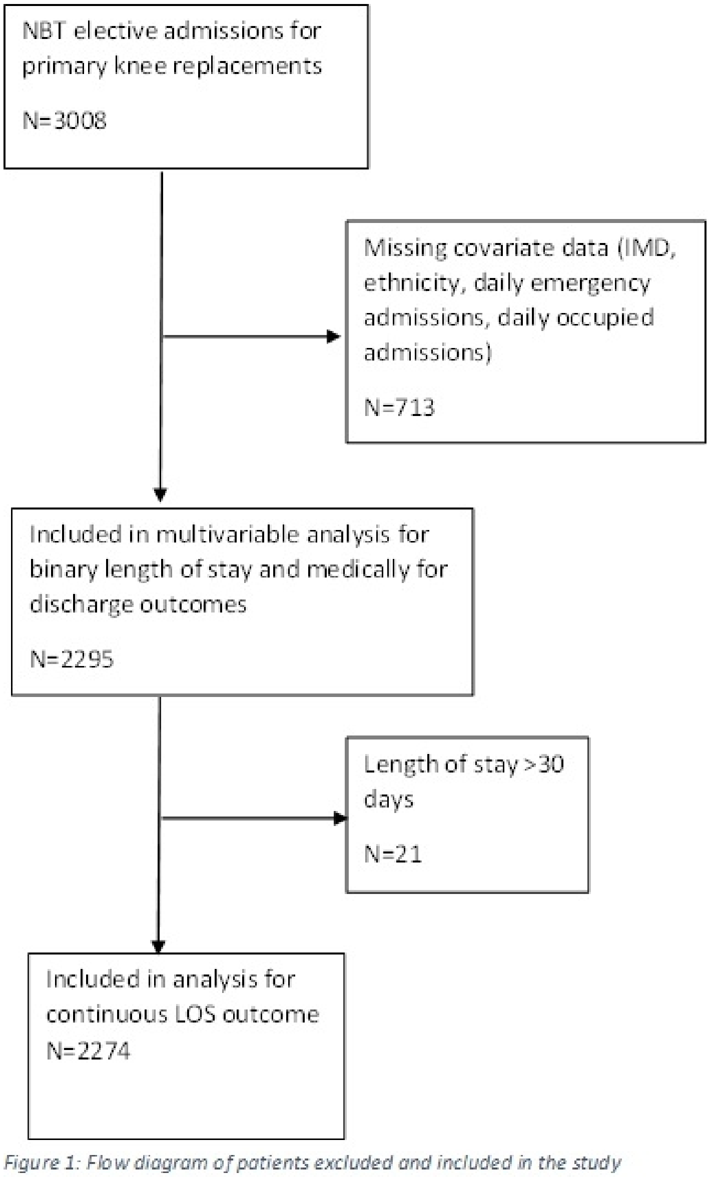

Figure 1: Flow diagram of patients excluded and included in the study

102x172mm (96 x 96 DPI)

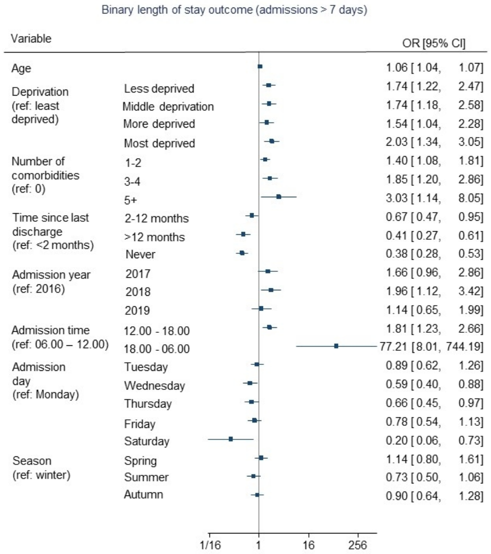

Figure 2: Predictors of length of stay of more than seven days for patients following knee replacement surgery

338x190mm (96 x 96 DPI)

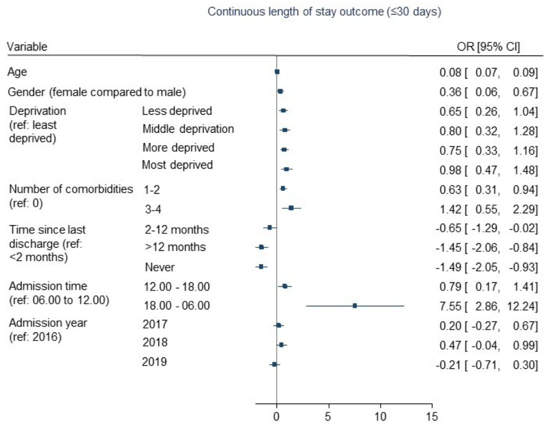

Figure 3: Predictors of length of stay (using a continuous measure) in patients following knee replacement surgery

338x190mm (96 x 96 DPI)

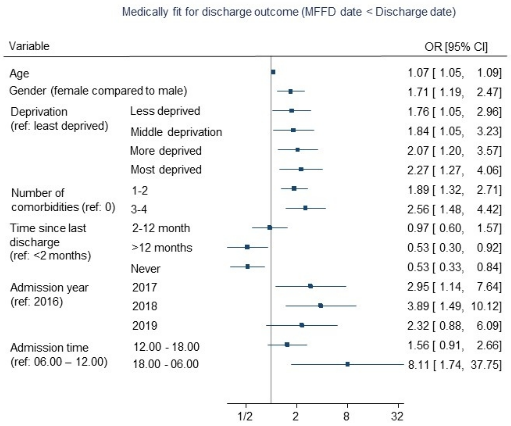

Figure 4: Predictors of remaining in hospital beyond being declared medically fit following knee replacement surgery

338x190mm (96 x 96 DPI)

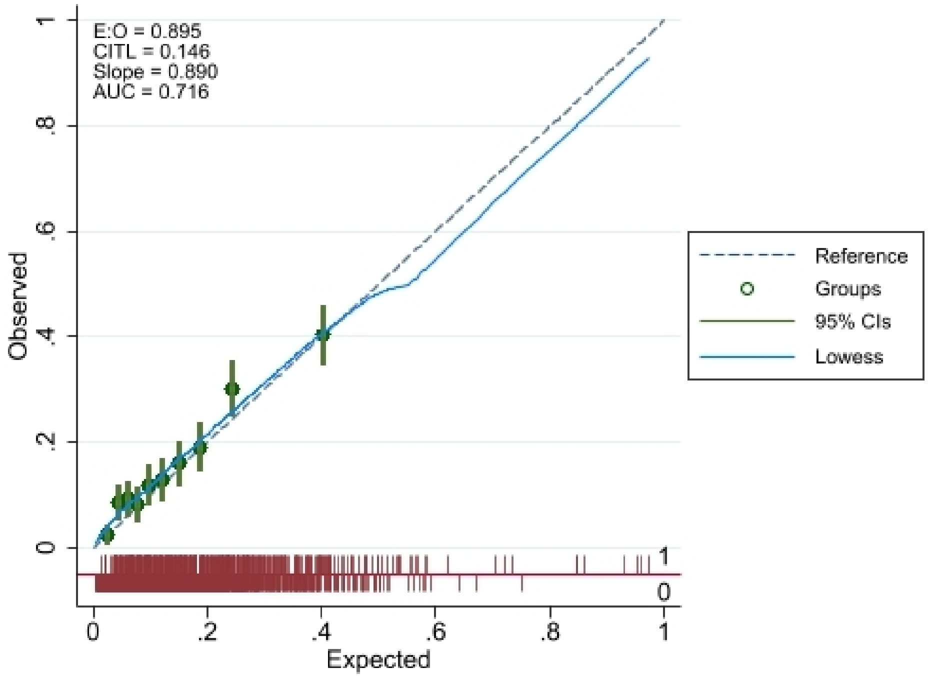

Figure 5: Calibration plot for final model for binary length of stay outcome

139x101mm (96 x 96 DPI)

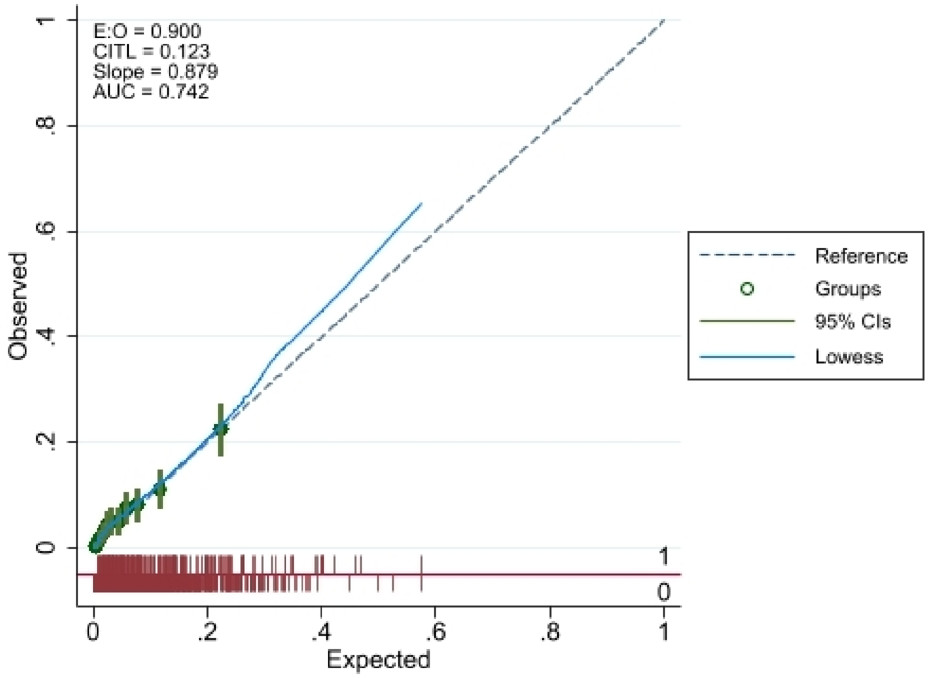

Figure 6: Calibration plot for final model for medically fit for discharge outcome

139x101mm (96 x 96 DPI)

Supplementary material

Supplementary Table S1: OPCS-4 codes used to identify primary hip and knee replacement operations

Supplementary Table S2: Sample characteristics for total sample (N=3008), those with complete data and with a previous admission (N=1221) and those with complete data and no previous admission (N=2295)

Supplementary Table S3: Multivariable models for binary length of stay outcome (admissions > 7 days)

Supplementary Figure S1: Calibration plot for binary length of stay outcome (full regression model)

Supplementary Figure S2: Calibration plot for binary length of stay outcome (backwards selection regression model)

Supplementary Table S4: Multivariable models for continuous length of stay outcome (≤ 30 days)

Supplementary Table S5: Multivariable models for medically fit for discharge date outcome (MFFD date < discharge date)

Supplementary Figure S3: Calibration plot for medically fit for discharge (full regression model)

Supplementary Figure S4: Calibration plot for medically fit for discharge (backwards selection regression model)

For peer review only

Supplementary Table S1: OPCS-4 codes used to identify primary hip and knee replacement operations

Category	Code	Description	Notes
Primary Total Knee Replacement	W40.1	Primary total prosthetic replacement of knee joint using cement	
	W40.8	Other specified total prosthetic replacement of knee joint using cement	
	W40.9	Unspecified total prosthetic replacement of knee joint using cement	
	W41.1	Primary total prosthetic replacement of knee joint not using cement	
	W41.8	Other specified total prosthetic replacement of knee joint not using cement	
	W41.9	Unspecified total prosthetic replacement of knee joint not using cement	
	W42.1	Primary total prosthetic replacement of knee joint NEC	
	W42.8	Other specified other total prosthetic replacement of knee joint	
	W42.9	Unspecified other total prosthetic replacement of knee joint	
	O18.1	Primary hybrid prosthetic replacement of knee joint using cement	
	O18.8	Other specified hybrid prosthetic replacement of knee joint using cement	
	O18.9	Unspecified hybrid prosthetic replacement of knee joint using cement	
Resurfacing / Reconstruction	W58.1	Primary resurfacing arthroplasty of joint	Require combination with site + combination codes to ID
	W58.8	Other specified reconstruction of joint	Require combination with site + combination codes to ID
	W58.9	Unspecified other reconstruction of joint	Require combination with site + combination codes to ID
Primary unicondylar / unicompartmental knee operations	W52.1	Primary prosthetic replacement of articulation of bone using cement NEC	Require combination with site + combination codes to ID
	W52.8	Other specified prosthetic replacement of articulation of other bone using cement	Require combination with site + combination codes to ID

For peer review only

W52.9	Unspecified prosthetic replacement of articulation of other bone using cement	Require combination with site + combination codes to ID
W53.1	Primary prosthetic replacement of articulation of bone not using cement NEC	Require combination with site + combination codes to ID
W53.9	Unspecified prosthetic replacement of articulation of other bone not using cement	Require combination with site + combination codes to ID
W54.0	Conversion from previous prosthetic replacement of articulation of bone NEC	Require combination with site + combination codes to ID
W54.1	Primary prosthetic replacement of articulation of bone NEC	Require combination with site + combination codes to ID
W54.8	Other specified other prosthetic replacement of articulation of other bone	Require combination with site + combination codes to ID
W54.9	Unspecified other prosthetic replacement of articulation of other bone	Require combination with site + combination codes to ID

For peer review only

Supplementary Table S2: Sample characteristics for total sample (N=3008), those with complete data and with a previous admission (N=1221) and those with complete data and no previous admission (N=2295)

Main characteristics		Total sample N=3008	Complete case sample included in multivariable model with continuous days since last discharge variable N=1221	Complete case sample included in multivariable model with categorical days since last discharge variable N=2295
Age, mean (SD)		67.5 (11.4)	68.0 (11.2)	67.4 (11.3)
Sex, N (%)	Female	1803 (59.9)	734 (60.1)	1381 (60.2)
	Male	1205 (40.1)	487 (39.9)	914 (39.8)
IMD score, N (%)	1 (least deprived)	753 (25.0)	310 (25.4)	588 (25.6)
	2	719 (23.9)	297 (24.3)	564 (24.6)
	3	515 (17.1)	225 (18.4)	395 (17.2)
	4	538 (17.9)	212 (17.4)	418 (18.2)
	5 (most deprived)	426 (14.2)	177 (14.5)	330 (14.4)
	(missing)	57 (1.9)	-	-
Ethnicity, N (%)	Non-White	90 (3.0)	47 (3.9)	76 (3.3)
	White	1827 (60.7)	779 (63.8)	1368 (59.6)
	(missing)	1091 (36.3)	395 (32.4)	851 (37.1)
Comorbidities, N (%)	0	1681 (55.9)	618 (50.6)	1260 (54.9)
	1-2	1126 (37.4)	485 (39.7)	867 (37.8)
	3-4	175 (5.8)	101 (8.3)	148 (6.5)
	>=5	26 (0.9)	17 (1.4)	20 (0.9)

For peer review only

Supplementary Table S3: Multivariable models for binary length of stay outcome (admissions > 7 days) (N=2295)

Variable	Multivariable logistic regression (no variable selection)		Lasso regression	Elastic net logistic regression	Ridge regression	Multivariable logistic regression (backwards selection)	
	**OR (95%CI)**	**p**	**OR**	**OR**	**OR**	**OR (95%CI)**	**p**
**Age at admission**	1.06 (1.04 to 1.07)	<0.001	1.78	1.77	1.70	1.06 (1.04 to 1.07)	<0.001
**Sex (female vs male)**	0.91 (0.71 to 1.17)	0.470	0.98	0.98	0.97	-	
**Deprivation (IMD score)**							
1 (least deprived)	*1.00*		*1.00*	*1.00*	*1.00*	*1.00*	
2	1.74 (1.22 to 2.48)	0.002	1.20	1.20	1.19	1.74 (1.22 to 2.47)	0.002
3	1.76 (1.19 to 2.60)	0.005	1.17	1.16	1.16	1.74 (1.18 to 2.58)	0.005
4	1.55 (1.04 to 2.29)	0.030	1.12	1.12	1.12	1.54 (1.04 to 2.28)	0.030
5 (most deprived)	2.05 (1.36 to 3.09)	0.001	1.21	1.21	1.20	2.03 (1.34 to 3.05)	0.001
**Comorbidities (weighted Charlson index)**							
0	*1.00*		*1.00*	*1.00*	*1.00*	*1.00*	
1-2	1.40 (1.08 to 1.81)	0.011	1.15	1.15	1.16	1.40 (1.08 to 1.81)	0.011
3-4	1.86 (1.20 to 2.87)	0.005	1.15	1.15	1.16	1.85 (1.20 to 2.86)	0.006
>=5	2.96 (1.11 to 7.90)	0.030	1.10	1.10	1.11	3.03 (1.14 to 8.05)	0.026
**Time since Last Discharge**							

For peer review only

0-2 months	*1.00*		*1.00*	*1.00*	*1.00*	*1.00*	
2-12 months	0.66 (0.47 to 0.94)	0.023	0.89	0.89	0.91	0.67 (0.47 to 0.95)	0.025
12 months or more	0.40 (0.27 to 0.60)	<0.001	0.75	0.75	0.77	0.41 (0.27 to 0.61)	<0.001
never	0.38 (0.27 to 0.53)	0.761	0.65	0.66	0.68	0.38 (0.28 to 0.53)	<0.001
**Emergency over elective admissions ratio**	0.65 (0.06 to 7.72)	0.736	-	-	0.98	-	
**NEL admissions**	1.00 (0.99 to 1.01)	0.729	-	-	1.03	-	
**NEL occupied beds**	0.999 (0.995 to 1.004)	0.761	1.01	1.01	1.03	-	
**Admission hour category**							
06.00-12.00	*1.00*		*1.00*	*1.00*	*1.00*	*1.00*	
12.00-18.00	1.82 (1.23 to 2.68)	0.003	1.17	1.17	1.16	1.81 (1.23 to 2.66)	0.003
18.00-06:00	76.72 (7.91 to 744.34)	<0.001	1.26	1.26	1.25	77.21 (8.01 to 744.20)	<0.001
**Year of Admission**							
2016	*1.00*		*1.00*	*1.00*	*1.00*	*1.00*	
2017	1.70 (0.92 to 3.17)	0.093	1.16	1.16	1.12	1.66 (0.96 to 2.86)	0.071
2018	2.07 (1.05 to 4.08)	0.036	1.24	1.24	1.19	1.96 (1.12 to 3.42)	0.018
2019	1.21 (0.54 to 2.70)	0.639	-	1.00	0.94	1.14 (0.65 to 1.99)	0.658
**Day of the Week of Admission**							
Sunday							
Monday	*1.00*		*1.00*	*1.00*	*1.00*	*1.00*	

For peer review only

Tuesday	0.88 (0.62 to 1.25)	0.479	-	1.00	0.98	0.89 (0.62 to 1.26)	0.497
Wednesday	0.59 (0.39 to 0.87)	0.008	0.87	0.87	0.86	0.59 (0.40 to 0.88)	0.009
Thursday	0.65 (0.44 to 0.96)	0.031	0.91	0.90	0.89	0.66 0.45 to 0.97)	0.033
Friday	0.76 (0.52 to 1.12)	0.164	0.95	0.95	0.93	0.78 (0.54 to 1.13)	0.185
Saturday	0.21 (0.05 to 0.79)	0.021	0.84	0.84	0.85	0.20 (0.06 to 0.73)	0.014
**Season of Admission**							
Winter (Dec-Feb)	*1.00*		*1.00*	*1.00*	*1.00*	*1.00*	
Spring (Mar-May)	1.11 (0.77 to 1.62)	0.571	1.07	1.07	1.08	1.14 (0.80 to 1.61)	0.479
Summer (Jun-Aug)	0.70 (0.45 to 1.10)	0.121	0.91	0.91	0.91	0.73 (0.50 to 1.06)	0.102
Autumn (Sep-Nov)	0.87 (0.59 to 1.29)	0.490	0.98	0.98	0.96	0.90 (0.64 to 1.28)	0.562
C-statistic	0.73 (0.71 to 0.76)					0.72 (0.69 to 0.74)	

For peer review only

Fig S1: Calibration plot for length of stay (full regression model)

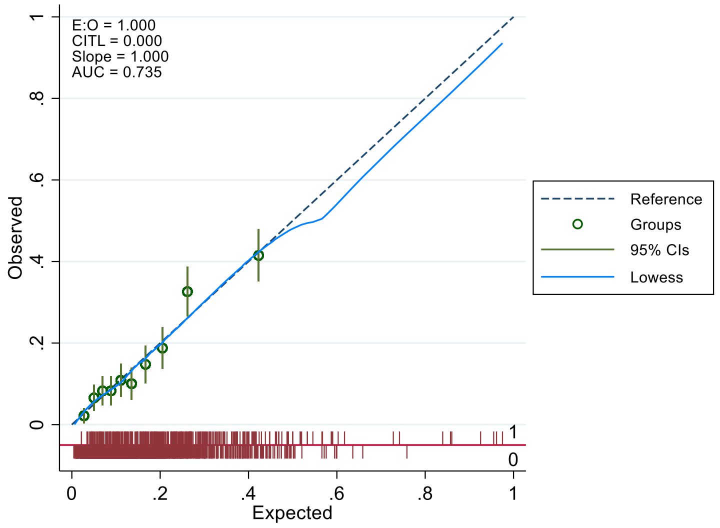

Fig S2: Calibration plot for length of stay (backwards selection regression model)

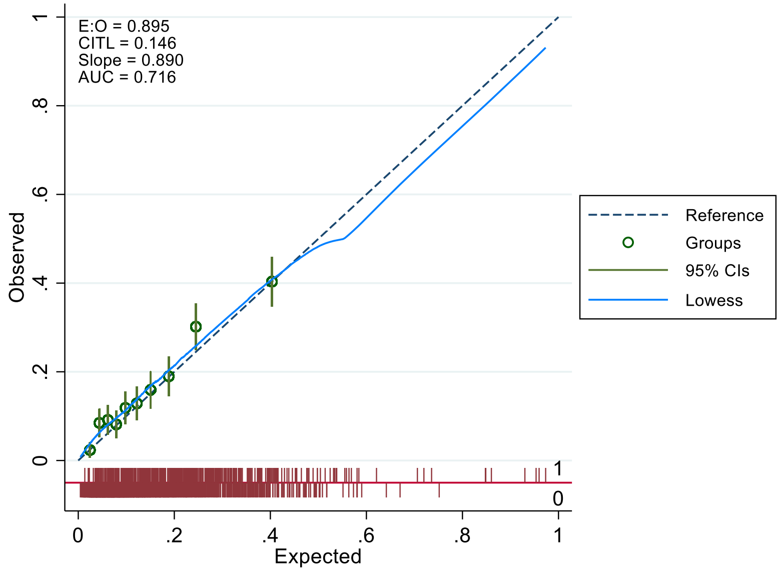

Supplementary Table S4: Multivariable models for continuous length of stay outcome (≤ 30 days) (N=2274)

Variable	Multivariable linear regression (no variable selection)		Lasso regression	Elastic net logistic regression	Ridge regression	Multivariable linear regression (backwards selection)	
	Coef (95%CI)	p	Coef	Coef	Coef	Coef (95%CI)	p
**Age at admission**	0.08 (0.07 to 0.09)	<0.001	0.87	0.87	0.83	0.08 (0.07 to 0.09)	<0.001
**Sex (female vs male)**	0.36 (0.05 to 0.66)	0.021	0.15	0.15	0.17	0.36 (0.06 to 0.67)	0.018
**Deprivation (IMD score)**							
1 (least deprived)	*0.00*		*0.00*	*0.00*	*0.00*	*0.00*	
2	0.62 (0.22 to 1.01)	0.002	0.18	0.18	0.20	0.65 (0.26 to 1.04)	0.001
3	0.81 (0.32 to 1.29)	0.001	0.22	0.22	0.24	0.80 (0.32 to 1.28)	0.001
4	0.73 (0.32 to 1.15)	0.001	0.20	0.20	0.22	0.75 (0.33 to 1.16)	<0.001
5 (most deprived)	0.96 (0.46 to 1.47)	<0.001	0.26	0.26	0.27	0.98 (0.47 to 1.48)	<0.001
**Comorbidities (weighted Charlson index)**							
0	*0.00*		*0.00*	*0.00*	*0.00*	*0.00*	
1-2	0.62 (0.31 to 0.93)	<0.001	0.27	0.27	0.28	0.63 (0.31 to 0.94)	<0.001
3-4	1.36 (0.50 to 2.23)	0.002	0.32	0.32	0.32	1.42 (0.55 to 2.29)	0.001
>=5	2.44 (0.12 to 4.75)	0.039	0.20	0.20	0.22	2.48 (0.15 to 4.81)	0.037
**Time since Last Discharge**							

For peer review only

0-2 months	*0.00*		*0.00*	*0.00*	*0.00*	*0.00*	
2-12 months	-0.65 (-1.29 to -0.01)	0.046	-0.15	-0.15	-0.13	-0.65 (-1.29 to -0.02)	0.044
12 months or more	-1.48 (-2.09 to -0.87)	<0.001	-0.46	-0.46	-0.42	-1.45 (-2.06 to -0.84)	<0.001
never	-1.50 (-2.06 to -0.94)	<0.001	-0.62	-0.62	-0.57	-1.49 (-2.05 to -0.93)	<0.001
**Emergency over elective admissions ratio**	-0.19 (-3.34 to 2.95)	0.904	-	-	-0.001	-	
**NEL admissions**	-0.01 (-0.02 to 0.01)	0.732	-	-	-0.05	-	
**NEL occupied beds**	-0.0005 (-0.0066 to 0.0057)	0.884	-	-	0.03	-	
**Admission hour category**							
06.00-12.00	*0.00*		*0.00*	*0.00*	*0.00*	*0.00*	
12.00-18.00	0.87 (0.24 to 1.50)	0.007	0.20	0.20	0.21	0.79 (0.17 to 1.41)	0.012
18.00-06:00	7.61 (2.91 to 12.31)	0.002	0.40	0.40	0.40	7.55 (2.85 to 12.24)	0.002
**Year of Admission**							
2016	*0.00*		*0.00*	*0.00*	*0.00*	*0.00*	
2017	0.36 (-0.30 to 1.03)	0.282	0.05	0.05	0.07	0.20 (-0.27 to 0.67)	0.411
2018	0.73 (0.04 to 1.41)	0.037	0.17	0.17	0.21	0.47 (-0.04 to 0.99)	0.073
2019	0.07 (-0.82 to 0.96)	0.879	-0.10	-0.10	-0.08	-0.21 (-0.71 to 0.30)	0.425
**Day of the Week of Admission**							

For peer review only

Sunday	-	-					
Monday	*0.00*		*0.00*	*0.00*	*0.00*		
Tuesday	1.11 (-0.34 to 0.56)	0.637	-	-	0.02		
Wednesday	0.13 (-0.37 to 0.64)	0.604	-	-	0.03		
Thursday	0.20 (-0.26 to 0.66)	0.399	0.02	0.02	0.06		
Friday	0.53 (0.05 to 1.01)	0.032	0.14	0.14	0.18		
Saturday	-0.49 (-1.55 to -0.57)	0.365	-0.04	-0.04	-0.06		
**Season of Admission**							
Winter (Dec-Feb)	*0.00*		*0.00*	*0.00*	*0.00*		
Spring (Mar-May)	-0.13 (-0.62 to 0.36)	0.604	-	-	-0.02		
Summer (Jun-Aug)	-0.52 (-1.06 to 0.02)	0.060	-0.14	-0.14	-0.16		
Autumn (Sep-Nov)	-0.10 (-0.60 to 0.40)	0.700	-	-	-0.02		
R^2	0.136					0.130	

For peer review only

Supplementary Table S5: Multivariable models for binary medically fit for discharge date outcome (MFFD date < discharge date) (N=2275)

Variable	Multivariable logistic regression (no variable selection)		Lasso regression	Elastic net logistic regression	Ridge regression	Multivariable logistic regression (backwards selection)	
	**OR (95%CI)**	**p**	**OR**	**OR**	**OR**	**OR (95%CI)**	**p**
**Age at admission**	1.07 (1.05 to 1.09)	<0.001	2.13	2.11	1.95	1.07 (1.05 to 1.09)	<0.001
**Sex (female vs male)**	1.70 (1.17 to 2.45)	0.005	1.28	1.29	1.28	1.71 (1.19 to 2.47)	0.004
**Deprivation (IMD score)**							
1 (least deprived)	*1.00*		*1.00*	*1.00*	*1.00*	*1.00*	
2	1.78 (1.06 to 3.02)	0.031	1.22	1.24	1.19	1.76 (1.05 to 2.96)	0.033
3	1.89 (1.08 to 3.33)	0.026	1.22	1.23	1.18	1.84 (1.05 to 3.23)	0.032
4	2.12 (1.22 to 3.67)	0.007	1.28	1.29	1.24	2.07 (1.20 to 3.57)	0.009
5 (most deprived)	2.23 (1.24 to 4.01)	0.007	1.27	1.28	1.22	2.27 (1.27 to 4.06)	0.006
**Comorbidities (weighted Charlson index)**							
0	*1.00*		*1.00*	*1.00*	*1.00*	*1.00*	
1-2	1.91 (1.32 to 2.74)	0.001	1.35	1.35	1.33	1.89 (1.32 to 2.71)	0.001
3-4	2.40 (1.37 to 4.22)	0.002	1.24	1.25	1.25	2.56 (1.48 to 4.42)	0.001
>=5	-		0.79	0.77	0.83	-	
**Time since Last Discharge**							
0-2 months	*1.00*		*1.00*	*1.00*	*1.00*	*1.00*	

For peer review only

2-12 months	0.94 (0.58 to 1.52)	0.792	-	1.00	1.01	0.97 (0.60 to 1.57)	0.907
12 months or more	0.48 (0.27 to 0.85)	0.012	0.79	0.79	0.82	0.53 (0.30 to 0.92)	0.024
never	0.51 (0.32 to 0.82)	0.005	0.74	0.74	0.76	0.53 (0.33 to 0.84)	0.007
**Emergency over elective admissions ratio**	0.97 (0.02 to 37.81)	0.986	-	-	1.03	-	
**NEL admissions**	0.99 (0.98 to 1.01)	0.230	0.91	0.85	0.87	-	
**NEL occupied beds**	1.00 (1.00 to 1.01)	0.592	1.05	0.07	1.09	-	
**Admission hour category**							
06.00-12.00	*1.00*		*1.00*	*1.00*	*1.00*	*1.00*	
12.00-18.00	1.61 (0.92 to 2.81)	0.096	1.13	1.15	1.14	1.56 (0.91 to 2.66)	0.103
18.00-06:00	8.81 (1.77 to 43.87)	0.008	1.14	1.14	1.14	8.11 (1.74 to 37.75)	0.008
**Year of Admission**							
2016	*1.00*		*1.00*	*1.00*	*1.00*	*1.00*	
2017	2.91 (1.00 to 8.41)	0.049	1.37	1.41	1.20	2.95 (1.14 to 7.64)	0.026
2018	4.37 (1.43 to 13.37)	0.010	1.57	1.64	1.36	3.89 (1.49 to 10.12)	0.005
	2.78 (0.79 to 9.82)	0.112	1.26	1.36	1.11	2.32 (0.88 to 6.09)	0.088
**Day of the Week of Admission**							
Sunday							
Monday	*1.00*		*1.00*	*1.00*	*1.00*		

For peer review only

Tuesday	1.67 (0.99 to 2.82)	0.057	1.19	1.19	1.16		
Wednesday	1.32 (0.75 to 2.32)	0.337	1.08	1.08	1.07		
Thursday	1.04 (0.57 to 1.88)	0.901	-	-	0.99		
Friday	1.68 (0.96 to 2.93)	0.069	1.18	1.19	1.17		
Saturday	0.65 (0.16 to 2.68)	0.548	0.96	0.94	0.95		
**Season of Admission**							
Winter (Dec-Feb)	*1.00*		*1.00*	*1.00*	*1.00*		
Spring (Mar-May)	1.37 (0.78 to 2.44)	0.276	1.11	1.12	1.13		
Summer (Jun-Aug)	1.38 (0.72 to 2.65)	0.327	1.10	1.12	1.13		
Autumn (Sep-Nov)	1.54 (0.85 to 2.76)	0.152	1.14	1.17	1.15		
C-statistic	0.77 (0.74 to 0.81)					0.74 (0.71 to 0.78)	

For peer review only

Fig S3: Calibration plot medically fit for discharge (full regression model)

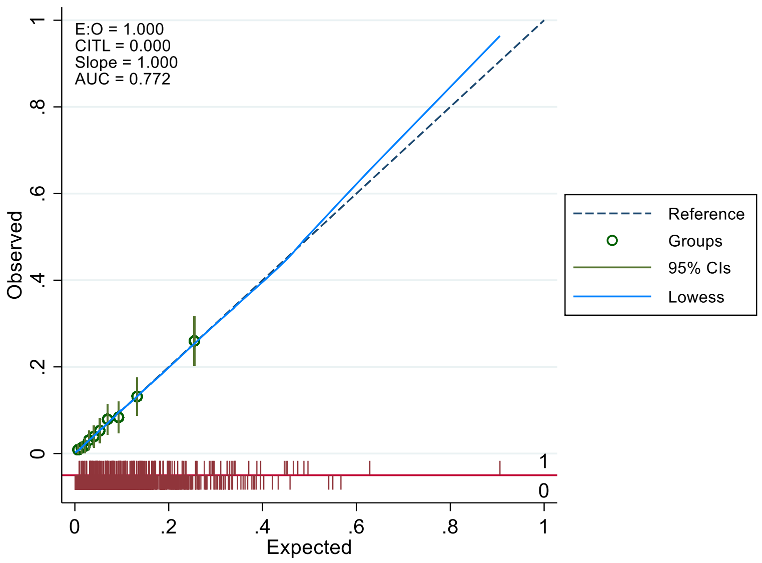

Fig S4: Calibration plot medically fit for discharge (backwards selection model)

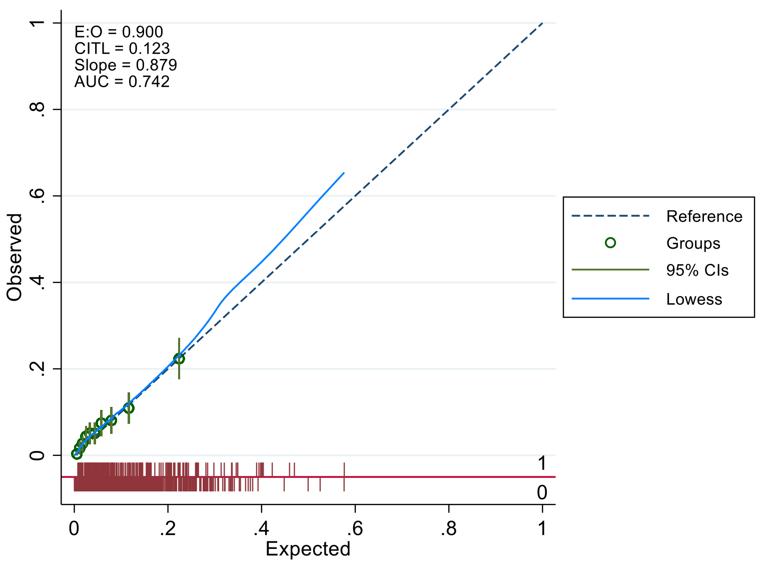

STROBE Statement—checklist of items that should be included in reports of observational studies

	Item No.	Recommendation	Page No.	Relevant text from manuscript
**Title and abstract**	1	(*a*) Indicate the study's design with a commonly used term in the title or the abstract	1	
		(*b*) Provide in the abstract an informative and balanced summary of what was done and what was found	1	
**Introduction**				
Background/rationale	2	Explain the scientific background and rationale for the investigation being reported	5-6	
Objectives	3	State specific objectives, including any prespecified hypotheses	6	"The aim of this study was to identify risk factors associated with prolonged length of hospital stay and staying in hospital longer than medically necessary following primary knee replacement surgery using linked routinely collected data from an NHS Trust in South-West England."
**Methods**				
Study design	4	Present key elements of study design early in the paper	7	"This is a longitudinal observational study"
Setting	5	Describe the setting, locations, and relevant dates, including periods of recruitment, exposure, follow-up, and data collection	7	Data sources section
Participants	6	(*a*) *Cohort study*—Give the eligibility criteria, and the sources and methods of selection of participants. Describe methods of follow-up<br>*Case-control study*—Give the eligibility criteria, and the sources and methods of case ascertainment and control selection. Give the rationale for the choice of cases and controls<br>*Cross-sectional study*—Give the eligibility criteria, and the sources and methods of selection of participants	7	

For peer review only - http://bmjopen.bmj.com/site/about/guidelines.xhtml

		(*b*) *Cohort study*—For matched studies, give matching criteria and number of exposed and unexposed<br>*Case-control study*—For matched studies, give matching criteria and the number of controls per case	N/A	
Variables	7	Clearly define all outcomes, exposures, predictors, potential confounders, and effect modifiers. Give diagnostic criteria, if applicable	7-8	Outcome variables & Predictive factors sections
Data sources/ measurement	8*	For each variable of interest, give sources of data and details of methods of assessment (measurement). Describe comparability of assessment methods if there is more than one group	7-8	Data sources, Outcome variables & Predictive factors sections
Bias	9	Describe any efforts to address potential sources of bias	8	"To explore bias in data availability, descriptive statistics for key patient characteristics (age, sex, socioeconomic deprivation, ethnicity and comorbidities) were summarised for three groups: all patients, those with complete case data, and those who had a previous discharge."
Study size	10	Explain how the study size was arrived at	7/8	"The Trust's electronic health records (EHR) were used to identify elective knee surgeries, using a combination of OPCS4 procedure and surgical site codes"<br>"Complete case analysis was used throughout. The complete case sample was n=2295, or 76.3% of the whole sample."

Continued on next page

For peer review only

Quantitative variables	11	Explain how quantitative variables were handled in the analyses. If applicable, describe which groupings were chosen and why	7-8	Outcome variables & Predictive factors sections
Statistical methods	12	(*a*) Describe all statistical methods, including those used to control for confounding	8-9	Statistical analysis section
		(*b*) Describe any methods used to examine subgroups and interactions	8-9	Statistical analysis section
		(*c*) Explain how missing data were addressed	8-9	Statistical analysis section
		(*d*) *Cohort study*—If applicable, explain how loss to follow-up was addressed<br>*Case-control study*—If applicable, explain how matching of cases and controls was addressed<br>*Cross-sectional study*—If applicable, describe analytical methods taking account of sampling strategy		
		(*e*) Describe any sensitivity analyses	8-9	Statistical analysis section
**Results**				
Participants	13*	(a) Report numbers of individuals at each stage of study—eg numbers potentially eligible, examined for eligibility, confirmed eligible, included in the study, completing follow-up, and analysed	10	"Between January 2016 and December 2019, 3008 primary elective knee replacements were performed. Of these, 2295 (76.3%) patients had data available for all variables and were included in the complete case analysis (see Figure 1). As the continuous length of stay outcome measure was capped at 30 days, the multivariable analyses for this outcome included 2274 patients"
		(b) Give reasons for non-participation at each stage		
		(c) Consider use of a flow diagram	Fig 1	
Descriptive data	14*	(a) Give characteristics of study participants (eg demographic, clinical, social) and information on exposures and potential confounders	Table 1	
		(b) Indicate number of participants with missing data for each variable of interest	Table 1	
		(c) *Cohort study*—Summarise follow-up time (eg, average and total amount)		
Outcome data	15*	*Cohort study*—Report numbers of outcome events or summary measures over time		
		Case-control study—Report numbers in each exposure category, or summary measures of exposure		

For peer review only - http://bmjopen.bmj.com/site/about/guidelines.xhtml
For peer review only

		Cross-sectional study—Report numbers of outcome events or summary measures	10	"The mean length of stay for patients who were in hospital for 30 days or less was 5.0 days (SD = 3.9), 15.4% of patients stayed in hospital for more than 7 days. Most patients (92.9%) did not remain in hospital once they were medically fit for discharge, but 7.1% did have a recorded medically fit for discharge date which preceded their discharge date."
Main results	16	(*a*) Give unadjusted estimates and, if applicable, confounder-adjusted estimates and their precision (eg, 95% confidence interval). Make clear which confounders were adjusted for and why they were included	Table 2	
		(*b*) Report category boundaries when continuous variables were categorized		
		(*c*) If relevant, consider translating estimates of relative risk into absolute risk for a meaningful time period		

Continued on next page

For peer review only

Other analyses	17	Report other analyses done—eg analyses of subgroups and interactions, and sensitivity analyses	Supplementary materials	
**Discussion**				
Key results	18	Summarise key results with reference to study objectives	11	Summary of main findings section
Limitations	19	Discuss limitations of the study, taking into account sources of potential bias or imprecision. Discuss both direction and magnitude of any potential bias	12	Strengths and limitations section
Interpretation	20	Give a cautious overall interpretation of results considering objectives, limitations, multiplicity of analyses, results from similar studies, and other relevant evidence	13	Implications for practice section
Generalisability	21	Discuss the generalisability (external validity) of the study results	12	Strengths and limitations section
**Other information**				
Funding	22	Give the source of funding and the role of the funders for the present study and, if applicable, for the original study on which the present article is based	3 & 9	Funding section & Role of funding sections

*Give information separately for cases and controls in case-control studies and, if applicable, for exposed and unexposed groups in cohort and cross-sectional studies.

Note: An Explanation and Elaboration article discusses each checklist item and gives methodological background and published examples of transparent reporting. The STROBE checklist is best used in conjunction with this article (freely available on the Web sites of PLoS Medicine at http://www.plosmedicine.org/, Annals of Internal Medicine at http://www.annals.org/, and Epidemiology at http://www.epidem.com/). Information on the STROBE Initiative is available at www.strobe-statement.org.

For peer review only - http://bmjopen.bmj.com/site/about/guidelines.xhtml
